# Inelastic scattering dynamics of hyperthermal O atoms on engineering surfaces relevant to satellites in low Earth orbit

**DOI:** 10.1007/s12567-025-00628-5

**Published:** 2025-06-27

**Authors:** Chenbiao Xu, Adriana Caracciolo, Pedro D. C. Jorge, Irina Gouzman, Marcin D. Pilinski, Timothy K. Minton

**Affiliations:** 1 Ann and H.J. Smead Department of Aerospace Engineering Sciences, University of Colorado, Boulder, CO 80303 USA; 2 Space Environment Department, Soreq NRC, 81800 Yavne, Israel; 3 Laboratory for Atmospheric and Space Physics, University of Colorado Boulder, Boulder, CO 80303 USA

**Keywords:** Atomic oxygen, Scattering dynamics, Molecular beam, Satellite drag

## Abstract

Understanding the gas-surface momentum transfer of oxygen atoms, a major component of the residual atmosphere at very low Earth orbit (VLEO) altitudes of 100–450 km, on materials on the exterior of satellites is important for estimating drag. We have thus investigated the scattering dynamics of orbital-velocity O atoms on four representative materials fluorinated ethylene propylene (FEP) polymer, aluminum with a chromate conversion Alodine coating (Al), solar cell cover glass with a MgF_2_ coating (CG), and a glass-reinforced epoxy laminate circuit board material (FR4). A pulsed hyperthermal atomic-oxygen beam with a nominal translational energy of ~ 4.7 eV was directed at the target surface, and the scattered products were detected with a rotatable mass spectrometer. Time-of-flight (TOF) distributions were measured with various incident beam angles (*θ*_*i*_ = 60°, 45°, 30°, 15°, 0°) for O atoms scattered in and out of the plane defined by the incident beam and surface normal. For both in-plane and out-of-plane scattering experiments, TOF distributions of O atoms exhibited mostly impulsive scattering, with atom-surface scattering events occurring on a time scale too short for thermal equilibrium to be achieved. A small fraction of thermally desorbed O atoms was also observed. In addition, both the flux and energy of the scattered O atoms were found to be higher when exiting the CG and FEP surfaces than when exiting the FR4 and Al surfaces. The lower flux of O atoms scattering from FR4 and Al at a given final angle (*θ*_*f*_), is the result of the combined effects of reactive collisions leading to OH and H_2_O products and the multiple-bounce trajectories of the incident O atoms on the rough surfaces, which leads to scattering angle randomization. Characterization of the material surfaces was consistent with the observed scattering dynamics: CG and FEP surfaces are relatively smooth at the nanometer scale, while FR4 and Al surfaces are comparatively rough. Regardless of surface roughness, the average fractional energy transfer to the surface is well parameterized by the angle through which the incident O atoms were deflected as they scattered from the surface. The fraction of thermally desorbed O atoms tends to be higher for the FR4 and Al surfaces than for the CG and FEP surfaces, which is consistent with greater energy accommodation on the FR4 and Al surfaces. The results suggest that FR4 and Al surfaces will lead to increased drag compared to CG and FEP surfaces, as a result of the greater overall energy accommodation of incident O atoms on the rougher surfaces.

**Supplementary Information:**

The online version contains supplementary material available at 10.1007/s12567-025-00628-5.

## Introduction

Satellites play a central role in various fields such as communications, remote sensing and Earth observation, navigation and positioning, and weather forecasting [1]. Satellite materials must withstand the challenging conditions present in low Earth orbit (LEO), including atomic oxygen (AO), temperature variations, electromagnetic radiation, outgassing, and micrometeoroids and orbital debris. These factors can significantly alter the morphology of satellite materials and damage their surfaces through oxidation, ablation, cracking, pitting, and contamination. Additionally, depending on altitude, the density of the atmosphere at LEO altitudes may cause non-negligible drag through momentum exchange between atmospheric atoms and molecules and satellite surfaces, leading to a reduction in orbital energy. Therefore, drag estimation is crucial for orbital path determination and collision avoidance analysis [2, 3], as well as lifetime prediction.

Furthermore, there is a growing interest in the utilization of very low Earth orbit (VLEO, 100–450 km in altitude). Operating satellites at lower altitudes in VLEO offers several benefits, including enhanced image resolution, improved data transfer rates, and cost reduction in satellite development and launching [4, 5]. Recent missions, such as GOCE, SLATS, and SOAR, have specifically focused on studying and operating satellites at progressively lower altitudes within the VLEO region, and these missions have helped to prepare the way for future VLEO satellites that could take advantage of the benefits of VLEO operation [6–9]. However, VLEO altitudes present more challenging conditions compared to higher LEO altitudes. The denser atmosphere in the VLEO region requires the use of materials that can resist the especially corrosive effects of a high flux of AO. In addition, the higher atmospheric density requires the use of a propulsion system to maintain orbit, resulting in increased mass and design complexity. Consequently, accurate drag prediction in VLEO plays an essential role in planning and operating in this challenging region of space. Drag prediction is not only important for orbit determination but also for managing and reducing aerodynamic drag through the identification of low-drag (and AO-resistant) materials that can extend the operational lifetime of VLEO satellites by minimizing propellant consumption [10].

Satellite drag coefficients have been modeled using different degrees of simplification in the description of gas-surface scattering. It has been argued that, for altitudes below 300 km, surfaces are completely covered with adsorbates, which promote high energy accommodation of incident molecules and diffuse scattering nearly independently of the surface material [11, 12]. Therefore, a model that assumes diffuse scattering with incomplete accommodation has been used to estimate satellite drag coefficients [13]. Moreover, the degree of accommodation of incident gas atoms or molecules with surfaces has been assumed to depend on surface coverage, which can be estimated using analytical models that depend on oxygen partial pressure [14]. More recently, the previous gas-surface interaction models have been compared with the Cercignani-Lampis-Lord scattering kernel, and significant differences were found in the computed drag coefficients and on the atmospheric properties derived from it, especially above 500 km [3, 15]. On the other hand, recent molecular beam-surface scattering experiments have shown non-diffuse (quasi-specular) scattering, with relatively low energy accommodation, for high incident angles even when the surface was covered with atomic oxygen [16].

Ultimately, modeling of gas-surface scattering dynamics is an important source of uncertainty in the computation of LEO and VLEO satellite drag coefficients [2]. For a particular gas system (defined by gas species and flow velocity), the scattering dynamics are not only controlled by measurable surface parameters like RMS roughness or the atomic arrangement of the surface, but they also depend on the interaction potential between the surface and the impinging energetic atom or molecule. Atoms can scatter predominantly in the forward, roughly specular, direction from surfaces that are rough on an atomic scale [17], while a highly ordered surface can promote scattering consistent with a corrugated gas-surface interaction potential [16, 18]. The scattering dynamics of O atoms on satellite materials in conditions relevant to LEO/VLEO environments are important for drag reduction, yet they remain poorly understood.

We have thus used a molecular beam-surface scattering technique to characterize the scattering dynamics of O atoms on representative satellite materials. The experiments provide translational energy and angular flux distributions of O atoms that scatter from the surface at different final polar and azimuthal angles (*θ*_*f*_ and *ϕ*_*f*_, respectively) for several incident angles (*θ*_*i*_), where the polar angles are referenced to the surface normal and the azimuthal angle is referenced to the plane containing the incident beam and surface normal. The scattering dynamics are therefore characterized in detail, which is crucial for drag prediction. The sample materials were selected based on two criteria. First, the materials should be commonly used on satellites and have exposure to the external environment. Second, the samples should exhibit degrees of roughness that span several orders of magnitude. The four samples chosen were: (1) fluorinated ethylene propylene (FEP), a copolymer of hexafluoropropylene and tetrafluoroethylene, used for thermal control; (2) MgF_2_-coated solar cell cover glass (CG), used on rigid solar cells; (3) glass-reinforced epoxy laminate circuit board material (FR4), used for electronics; (4) aluminum with an Alodine chromate conversion coating (Al), used as a structural material. This set of samples with disparate surfaces provides an opportunity to investigate O-atom scattering dynamics on practical engineering surfaces and gain insight into the factors that control energy transfer and scattering direction in energetic atom-surface collisions. Understanding these factors is necessary for the development of high-fidelity gas-surface models used for satellite design and drag estimation.

## Experimental methods

### Molecular beam-surface scattering apparatus

The experiments were performed with a crossed molecular beams apparatus with a rotatable mass spectrometer detector that was reconfigured for molecular beam-surface scattering experiments, as described in detail in previous publications [17, 19–21]. A hyperthermal pulsed O-atom beam with a 2 Hz repetition rate was generated using a laser-induced breakdown source [22]. A home-built piezoelectric valve injects a burst of pure molecular oxygen (O_2_, 99.99%), at a pressure of 38 bar, through a 1 mm dia. orifice into the 1 mm dia. throat of a conical nozzle. A CO_2_ TEA laser with a pulse energy of 7 J is focused into the O_2_ gas in the nozzle throat using a gold mirror with a 1 m radius of curvature, which induces a gas breakdown and the creation of a hot plasma. The ensuing blast wave dissociates and accelerates the gas. The expansion is complete before most gas particles can recombine, resulting in a beam with a nominal velocity of ~ 8 km s^−1^, consisting of ground-state oxygen atoms, O(^3^P), with a small component (~ 10%) of ground-state O_2_(${}^{3}\Sigma_{g}^{ - }$) [21, 23] and a negligible ionic component ($\ll 1\%$) [17].

The O/O_2_ hyperthermal beam was collimated through a 2 mm dia. skimmer placed 93 cm after the nozzle cone apex. A 1.3 mm wide × 2.8 mm high aperture was placed 3.5 cm downstream from the skimmer to collimate the beam further. A synchronized chopper wheel (17.8 cm dia.) with three equally spaced 1.5 mm wide slots and a rotation speed of 300 Hz was used to select a narrow range of beam velocities from the beam pulse. The chopper wheel blocked the light emitted by the plasma as well as the residual ions, which have velocities higher than the O and O_2_ in the hyperthermal beam.

**Fig. 1:**
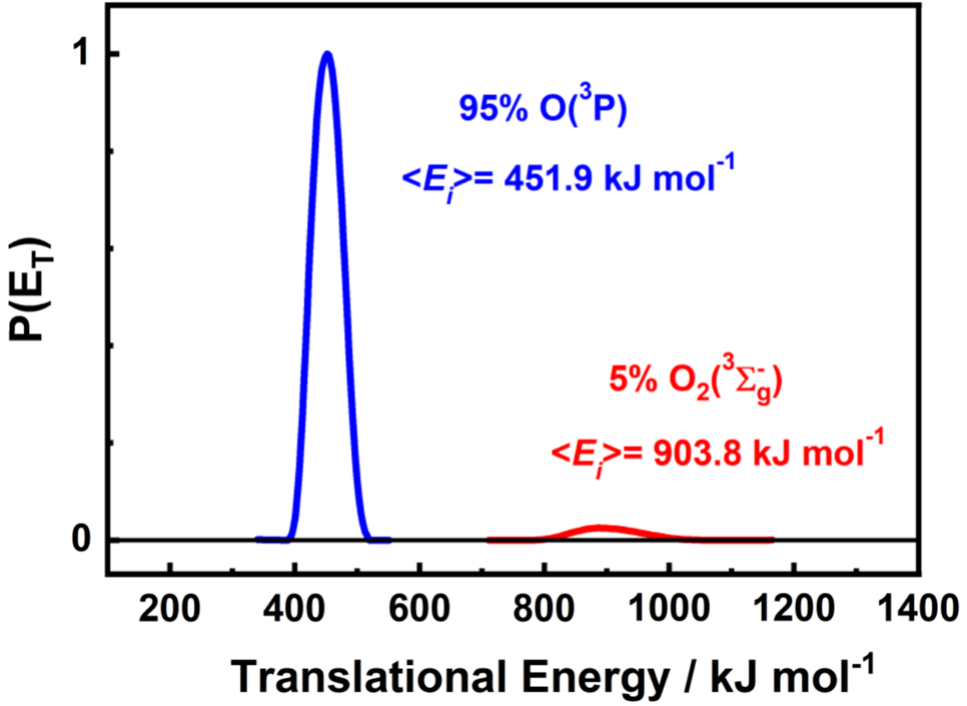
Translational energy distributions of the O and O_2_ components of the hyperthermal beam. The red and the blue curves represent the O and O_2_ distributions after velocity selection by a chopper wheel. The areas of the translation energy distributions of O and O_2_ are proportional to their mole fractions in the hyperthermal beam

Figure 1 shows the translational energy distributions, *P(E*_*T*_*)*, of O and O_2_ in the hyperthermal beam after velocity selection by the chopper wheel, as well as the mole fractions of these two components of the beam. To obtain the translational energy distributions, the beam was directed into the mass spectrometer equipped with an electron-impact ionizer, a quadrupole mass filter, and a continuous dynode electron multiplier, where the electron multiplier was operated in pulse counting mode. Number density distributions, *N(t)*, of the incident beam pulse were obtained as a function of the flight time over the known distance from the nozzle throat to the electron-impact ionizer (132.9 cm). These *N(t)* distributions, also referred to as time-of-flight (TOF) distributions were used to derive velocity and translational energy distributions of the O atoms and O_2_ molecules that passed through the ionizer, under the assumption that the beam originated from a point source at the nozzle throat. After correcting for the relative ionization cross sections of O and O_2_ (1:1.9) [21], the beam was found to consist of 95% O atoms and 5% O_2_ molecules. The average translational energy of the O atoms was 〈*E*_*i*_〉 = 451.9 kJ mol^−1^, with a full width at half maximum (FWHM) of 63 kJ mol^−1^*.* The average translational energy of the O_2_ molecules was 〈*E*_*i*_〉  = 903.8 kJ mol^−1^, with a FWHM = 132 kJ mol^−1^.

TOF distributions of scattered products were collected with the rotatable mass spectrometer detector following impingement of the incident hyperthermal beam pulse on the target surface. To obtain a given TOF distribution, the relative number density of the scattered products having a selected mass-to-charge ratio, *m/z*, was measured as a function of the flight time from the surface to the electron-impact ionizer of the mass spectrometer, a distance of 34.4 cm, and collection of a given TOF distribution typically involved the accumulation of these *N(t)* distributions for several hundred pulses of the incident beam. The TOF distributions were acquired with various sets of incident (*θ*_*i*_) and final (*θ*_*f*_*, ϕ*_*f*_) angles, where the polar angles (*θ*_*i*_, *θ*_*f*_) are referenced to the surface normal and the azimuthal angle (*ϕ*_*f*_) is referenced to the plane defined by the incident beam direction and the surface normal. A corresponding translational energy distribution was derived from each TOF distribution, from which average energy and total relative flux were obtained.

### Samples

The samples used in this work were procured from commercial manufacturers and were intended to be representative of satellite materials currently in use.

#### Solar cell cover glass (CG)

Cover glass material is used as a top layer on rigid solar cells, where the CG transparency allows sunlight to reach the semiconductor underneath while also providing thermal insulation and filtering of ultraviolet (UV), infrared (IR), electron, and proton radiation. The CG sample was produced by Qioptic, labeled as “CMG 150 A/R Coated Coverglass,” and is a cerium-doped borosilicate glass with a MgF_2_ coating.

#### Fluorinated ethylene propylene (FEP)

FEP is a clear material that is a copolymer of hexafluoropropylene and tetrafluoroethylene. This material was chosen because it is commonly used on the external surfaces of satellites for thermal control [24, 25], and it is also relatively AO-resistant [26]. The sample surface was the uncoated top layer of a space-grade FEP film tape. The FEP film had a silver coating and pressure-sensitive adhesive on the back side (a Sheldahl product purchased from Bron Aerotech).

#### Composite printed circuit board (FR4)

This material was selected because it is commonly used in satellite electronics. PCBs are characterized by copper circuits printed on a fiber glass core. The sample chosen is referred to as flame retardant, FR4, and it has an epoxy and carbon-based outer layer (solder mask). Although FR4 generally does not exceed 30% of the satellite surface, it was observed that the color of the solder mask (green, in the case of FR4) can alter the average temperature of the overall satellite. Green and black solder masks were found to increase the satellite body average temperature by 16 K compared to white PCBs [27]. The sample was extracted from a commercial printed circuit board by cutting a piece that excluded the copper components; thus, the relevant surface was the solder mask layered on the top of the composite glass-reinforced epoxy laminate material.

#### Aluminum with an Alodine chromate conversion coating (Al)

This material was selected because of its use for satellite structural components and communication systems. Aluminum is advantageous due to its light weight and high mechanical resistance. To enhance the material’s chemical resistance, aluminum is chemically converted with chromate to create a protective coating on the outer layer of the metallic surface that may be exposed to AO [28, 29]. The Alodine-treated aluminum sample was provided by Montana State University’s Space Science and Engineering Laboratory and it was part of a ready-to-fly antenna bracket.

### Surface characterization

Scanning electron microscopy (SEM), energy dispersive X-ray spectroscopy (EDS), and X-ray photoelectron spectroscopy (XPS) were used to analyze sample surfaces before and after scattering experiments. SEM measurements were carried out using a Zeiss SUPRA 55VP Field Emission SEM (Carl Zeiss AG, Germany) instrument in a high-vacuum mode. The samples were analyzed using a low-voltage electron beam, set at 1 kV, to prevent charging of the surface. XPS measurements were performed using a PHI 5600 (PHI, USA) instrument equipped with an Al Kα monochromatic source (1486.6 eV). Sample charging was compensated with a charge neutralizer, and the position of the C 1s core level line at 285.0 eV was taken as an energy reference for all the peaks for FR4, Al, and CG. Because of the negligible carbon signal observed from the FEP surface, the F 1 s core level line at 686 eV was used as an energy reference for this material. All XPS spectra were collected with a pass energy of 46.95 eV. For quantitative analysis, PHI relative sensitivity factors (RSF) were used: F 1 s = 1, O 1 s = 0.711, C 1 s = 0.296, N 1 s = 0.477, Cr 2p_3_ = 1.583, and Sn 3d_5_ = 4.725. The elemental composition of the samples was also evaluated with an Oxford Instruments EDS system, attached to a SU3500 Hitachi Tungsten filament SEM with 30 keV primary electron beam energy. While XPS is sensitive to the top 10 nm surface layer, EDS provides elemental information on the micrometer length scale.

To determine the roughness of the target surfaces, the surface morphology was probed by atomic force microscopy (AFM) and white-light interferometry. The CG and FEP sample surfaces were examined using a NaniteAFM instrument from Nanosurf AG, Switzerland, operated with an Easyscan 2 controller and Nanosurf software. All images were obtained in tapping mode using Aspire™ conically-shaped silicon tips, model CT170R, with a resonant frequency of approximately 170 kHz. The surface roughness was based on at least four measurements of 5 × 5 µm^2^ areas. The AFM images were processed using WSxM 4.0 software. A Veeco Model Wyko NT3300 white light interferometer was used to characterize the roughness of the FR4 and Al samples. All samples were analyzed without surface preparation, either as received or as they were when removed from the high vacuum scattering chamber.

### Experimental procedure

The samples were cleaned with isopropanol (99%) and then mounted in the high-vacuum scattering chamber (with a pressure of ~ 10^−7^ Torr maintained during experiments) on the heated copper plate of a sample holder, where they were held at an elevated temperature to remove some of the physisorbed molecular contaminants, such as H_2_O. A cartridge heater was inserted into a hole in the plate, allowing the plate to be heated from room temperature to ~ 700 K, although temperatures of 353–373 K were typically used. Even with the use of an oil-free turbomolecular pump and a cryopump to pump the scattering chamber, the low chamber pressure of ~ 10^−7^ Torr maintained during experiments, and the elevated sample temperatures, the samples may have had adsorbed hydrocarbons on their surfaces during an experiment, which might have come from adventitious carbon from sample handling in the laboratory or, less likely, from contamination in the chamber. The steady flux of O atoms onto the surface during an experiment (estimated to be ~ 10^14^ atoms cm^2^ s^−1^) would be expected to reduce hydrocarbon contamination on surfaces, though possibly not eliminating it. In addition, the bombardment of the surfaces by O atoms would be expected to result in a steady-state oxygen surface coverage, similar but probably not identical to that on leading surfaces in the LEO/VLEO environment. The surface chemistry was not characterized in situ during the experiments, so it is not possible to know the exact surface conditions during the beam-surface scattering experiments.

To ensure that data acquisition was performed with the sample surfaces at a steady state, TOF distributions of inelastically scattered O atoms were measured at an arbitrary *θ*_*f*_ until they no longer changed. Then, TOF distributions were acquired for O and O_2_ products at *m/z* = 16(O^+^) and 32(O_2_^+^). Data collection for a given sample typically proceeded for at least 12 h before the sample was replaced or its configuration was changed. The TOF distributions of scattered O atoms did not change during this time, suggesting that a steady-state was attained for each experimental run. The raw data were corrected for the ion flight time, IFT = *α* (*m/z*)^1/2^, where *m* is the detected product mass in atomic mass units, *z* is the ion charge, and the parameter, *α*, was determined empirically to be *α* = 2.47. In addition, the TOF distributions obtained for scattered O atoms were corrected for the dissociative ionization of O_2_ to O^+^ in the electron-impact ionizer. The fraction of the TOF distribution for O_2_, detected at *m/z* = 32, that appeared at *m/z* = 16 was determined to be 11%. This percentage was then used to scale the TOF distributions acquired at *m/z* = 32, and these scaled distributions were subtracted from the corresponding TOF distributions collected at *m/z* = 16 to yield the net O-atom TOF distributions. The TOF distributions of the O and O_2_ products were transformed into translational energy distributions, *P(E*_*T*_*)*, using the relation $P\left( {E_{T} } \right) \propto t^{2} N\left( t \right)$*.* The total relative product flux was obtained from the TOF distribution for a given set of incident and final angles by integration of the *P(E*_*T*_*)* distribution. An average final energy was also derived from each *P(E*_*T*_*)* distribution.

**Fig. 2:**
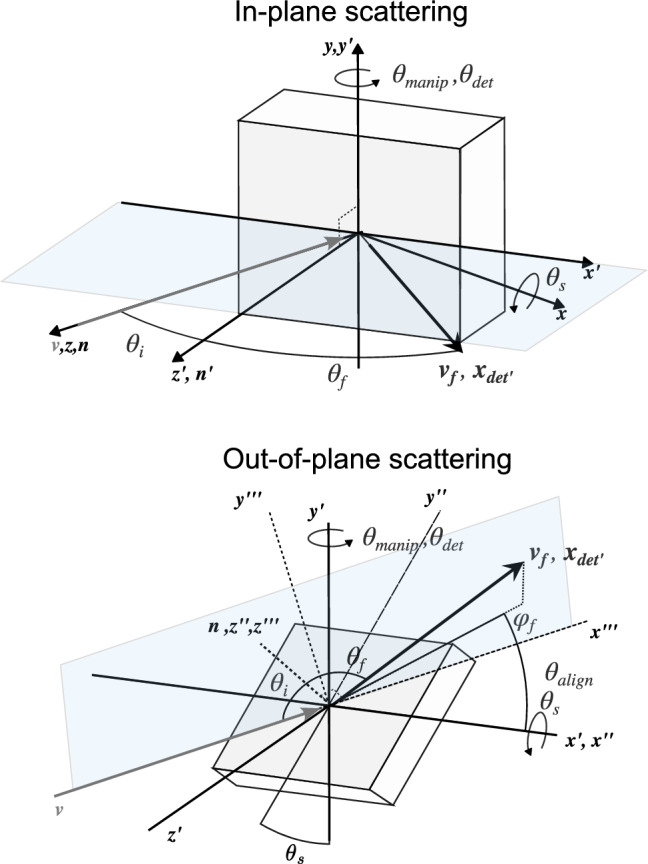
Schematic diagrams of the orientation of the target surface with respect to the incident beam for in-plane and out-of-plane scattering configurations. Rotated vectors are denoted with a prime symbol, with the number of primes indicating the number of rotaions

TOF distributions were collected at final angles in the plane of the incident beam and surface normal (“in-plane” scattering) with all sample surfaces, and TOF distributions were also collected at final angles that were out of the plane of the incident beam and surface normal (“out-of-plane” scattering) for the Al and FEP surfaces. The scattering angles are described in a spherical coordinate system (refer to Fig. 2). In the experiment, the incident beam direction was fixed, and the incident angle was adjusted by altering the surface orientation. Initially, the beam velocity $({\boldsymbol{v}}$) was aligned with the surface normal $\left( {\boldsymbol{n}} \right)$ such that ${\boldsymbol{v}} = v_{mag} \left( {0,0, - 1} \right)$ and ${\mathbf{n}} = \left( {0,0,1} \right)$***.*** The surface normal was reoriented about the *x*-axis using a sample mount tilted by an angle $\theta_{s}$ and about the *y*-axis by an adjustable angle $\theta_{manip}$. The detector is initially aligned with the rotated surface normal projected onto the *z-x* plane and has coordinates ${\boldsymbol{x}}_{{{\boldsymbol{det}}}} = l_{det} \left( {\sin \theta_{manip} ,0,\cos \theta_{manip} } \right)$. The detector was subsequently rotated by an angle $\theta_{det}$ about the *y*-axis (Fig. 2). Vectors subjected to rotation are denoted with a prime symbol, with the number of primes indicating the number of rotations. The incident polar angle is defined as the angle between the surface normal and the incident velocity vector. Similarly, the final polar angle is the angle between the surface normal and the detector position vector (aligned along the detection axis). These angles may be determined by applying the rotation matrix ${\boldsymbol{R}}_{{\boldsymbol{y}}} \left( {\theta_{det} } \right)$ to ${\boldsymbol{x}}_{{{\boldsymbol{det}}}}$ and by applying ${\boldsymbol{R}}_{{\boldsymbol{y}}} \left( {\theta_{manip} } \right){\boldsymbol{R}}_{{\boldsymbol{x}}} \left( {\theta_{s} } \right)$ to ${\boldsymbol{n}}$. The incident $\theta_{i} = \angle \left( {{\mathbf{vn}}^{\prime\prime}} \right)$ and final $\theta_{f} = \angle ({\mathbf{x}}_{{{\boldsymbol{det}}}} ^{\prime}{\mathbf{n}}^{\prime\prime})$ polar angles can be determined from the following equations,

1 $$\cos \theta_{{\mathrm{i}}} = \cos \theta_{s} \cos \theta_{{{\mathrm{manip}}}} ,$$

2 $$\cos \theta_{f} = \cos \theta_{s} \cos \theta_{{{\mathrm{det}}}} .{ }$$

For the in-plane configuration, the surface of the sample is perpendicular to the detector rotational plane (*θ*_*s*_ = 0), and *θ*_*i*_ and *θ*_*f*_ are equal to *θ*_*manip*_ and *θ*_*det*_, respectively. For the out-of-plane configuration, both *θ*_*i*_ and *θ*_*f*_ depend on *θ*_*s*_. We define the final azimuthal angle *ϕ*_*f*_ = 0, as being oriented in the plane defined by the surface normal and the incident velocity. *ϕ*_*f*_, is, in general, non-zero and can be determined from the detector’s position within a reference frame aligned with the incident velocity and the surface normal. In this transformed reference frame, the *z*-axis coincides with the rotated surface normal, and the *x*- and *y*-axes are oriented such that the incident velocity vector can be expressed with two components, ${\boldsymbol{v'''}} = \left( {{v_t},0,{v_n}} \right)$. The desired reference frame is obtained through a sequence of reference frame rotations. First about ${\boldsymbol{y}}$ by an angle *θ*_manip_, then about $\boldsymbol{x'}$ by an angle *θ*_*s*_*,* and finally about ${\boldsymbol{z}}$**′′** by an angle *θ*_align_ determined so that $v''' = \left( {{v_t},0,{v_n}} \right)$*.* Through algebraic manipulation, we find $\tan \theta_{{{\mathrm{align}}}} = \frac{{ - {\text{ sin}}\theta_{s} }}{{\tan \theta_{{{\mathrm{manip}}}} }}$. The tangent of the azimuthal angle is given by the ratio of *y* and *x* detector coordinates in this rotated reference frame

3 $$\tan \phi_{f} = \frac{{ - \sin {\theta_{{{\mathrm{det}}}} }\sin {\theta_{{{\mathrm{align}}}} } + \sin {\theta_{s} }\cos {\theta_{{{\mathrm{det}}}} }\cos {\theta_{{{\mathrm{align}}}} }}}{{\sin {\theta_{{{\mathrm{det}}}} }\cos {\theta_{{{\mathrm{align}}}} } + \sin {\theta_{{{\mathrm{align}}}} }\sin {\theta_{s} }\cos {\theta_{{{\mathrm{det}}}} }}}$$

As mentioned above, a heated copper plate was used to maintain the samples at temperatures above ambient. Samples mounted in the in-plane configuration were pressed by the copper plate against a stainless-steel frame, such that the detector rotation axis passed through the sample surface. For the out-of-plane experiments, the Al and FEP samples were adhered directly to heated copper plates that were cut with faces at tilt angles of *θ*_*s*_ = 20° and 45°, and the sample surfaces were oriented such that the detector viewing region on the surface was coincident with region where the incident beam impacted the surface. The FEP sample was supplied with pressure-sensitive adhesive on the back side, and it was simply pressed onto the tilted copper face. The Al sample was attached by using an electrically conductive polyimide adhesive: Creative Materials Inc., 110–19, which was stored at − 85°C before usage. A primary layer of adhesive was applied separately to the sample surface and the copper face and cured for 30 min at 150 °C. The two pieces were attached together with a second layer of adhesive, and the whole assembly was cured for 1 h at 180° C. For both in-plane and out-of-plane experiments, the scattered O and O_2_ were detected over the full angular range accessible to the detector. TOF distributions of O and O_2_ products were accumulated for various total numbers of beam pulses with various incident angles and collected twice at each final (*θ*_det_) angle by first increasing and then decreasing the final angle in 5° increments. The two TOF distributions collected for each set of incident and final angles were added to give the total TOF distribution that was used in the data analysis. A summary of the data acquisition conditions is shown in Table 1.

**Table 1:** Summary of the data acquisition conditions for studies of the inelastic scattering dynamics of O on the four surfaces used. The deflection angle is the difference between 180° and the angle between the scattering atom’s incident and final directions

Surface	Surface temperature, *T*_*s*_ (K)	Incident angle, *θ*_*i*_ (°)	Total beam pulses, *m/z* = 16 (O^+^) ^d^	Final angle range,* θ*_*f*_ (°)	Azimuthal angle range, *ϕ*_*f*_ (°)	Deflection angle range, χ(°)
CG	373	60 ^a^	6800	0 – 80	0 – 0	120 – 40
		45 ^a^	6400	5 – 80	0 – 0	130 – 55
		30 ^a^	5200	20 – 80	0 – 0	130 – 70
		15 ^a^	4000	35 – 80	0 – 0	130 – 85
		0 ^a^	2800	50 – 80	0 – 0	130 – 100
		− 15 ^a,e^	1600	65 – 80	0 – 0	130 – 115
FR4	353	60 ^a^	6800	0 – 80	0 – 0	120 – 40
		45 ^a^	6400	5 – 80	0 – 0	130 – 55
		30 ^a^	5200	20 – 80	0 – 0	130 – 70
		15 ^a^	4000	35 – 80	0 – 0	130 – 85
		0 ^a^	2800	50 – 80	0 – 0	130 – 100
		− 15 ^a,e^	1600	65 – 80	0 – 0	130 – 115
FEP, Al	373	60 ^a^	6800	0 – 80	0 – 0	120 – 40
		45 ^a^	6400	5 – 80	0 – 0	130 – 55
		30 ^a^	5200	20 – 80	0 – 0	130 – 70
		15 ^a^	4000	35 – 80	0 – 0	130 – 85
		0 ^a^	2800	50 – 80	0 – 0	130 – 100
		− 15 ^a,e^	1600	65 – 80	0 – 0	130 – 115
		60 ^b^	5200	20 – 80	102.1 – 15.8	122.1 – 42.8
		45 ^b^	4800	25 – 80	72.6 – 25.0	123.5 – 59.5
		30 ^b^	4000	35 – 80	70.4 – 42.8	127.8 – 77.8
		60 ^c^	FEP:3200 Al:6400	45 – 80	125.3 – 45.4	135.0 – 59.2
		45 ^c^	FEP:1600 Al:3200	65 – 80	117.8 – 100.1	126.7 – 104.2

^(a)^In-plane scattering configuration

^(b)^Out-of-plane scattering configuration with a tilt angle of *θ*_*s*_ = 20°

^(c)^Out-of-plane scattering configuration with a tilt angle of *θ*_*s*_ = 45°

^(d)^Total beam pulses for *m/z* = 32(O_2_^+^) are double the total beam pulses for O, except for *θ*_*s*_ = 45°, when the TOF distributions of O_2_ products were collected for 18,000 and 10,000 beam pulses for *θ*_*i*_ = 60° and 45°, respectively

^(e)^A negative incident angle for the in-plane configuration indicates that scattered products were detected at angles on the same side of the surface normal as the incident beam

## Results

### Surface characterization

**Fig. 3:**
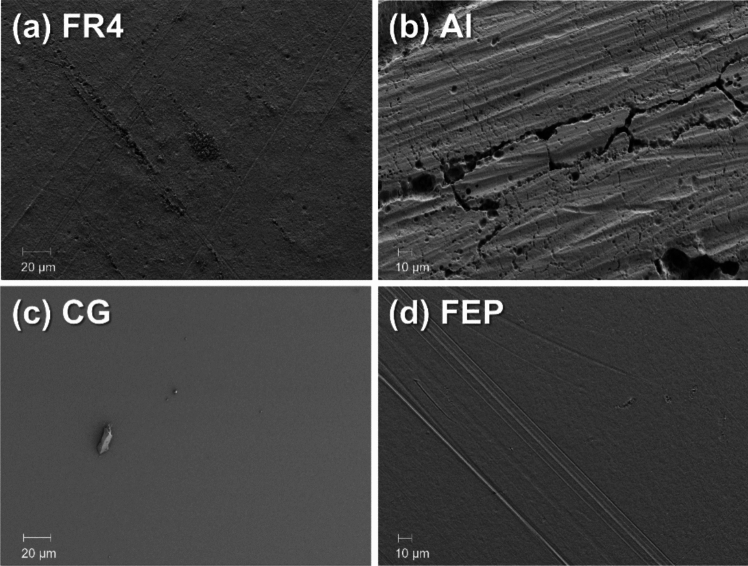
Representative SEM images of the surfaces of the as-received samples, collected with a relatively low magnification of 1000×. **a** FR4. **b** Al. **c** CG. **d** FEP

Low magnification (1000 ×) SEM images of the as received FR4, Al, CG, and FEP surfaces are shown in Fig. 3. The SEM images of the FR4, CG, and FEP samples show uniform surfaces, whereas the surface of the Al sample is nonuniform and exhibits significant defects and cracks over the whole surface. The CG and FEP surfaces are particularly smooth compared to those of FR4 and Al. SEM images of the CG surface show only traces of dust, while the FEP surface presents a few grooves, which are commonly generated during the fabrication process of the polymer.

The chemical compositions of the as-received sample surfaces were further investigated by EDS and XPS. EDS analysis of the FR4 sample showed that C and O atoms are the main elements (55.2 and 32.1%, respectively) by sampling the material in depth between 1 and 3 μm (Table 2). EDS analysis on the Al sample indicated that the main elements are Al (58.1%), followed by C (17.3%) and O (18.8%) in the same depth range (Table 3). XPS was used to determine the elemental chemical composition in the top ~ 10 nm of all four samples. XPS data (Table 2) acquired for FR4 showed high concentrations of C and O atoms (73.5 and 18.3%, respectively), which was qualitatively similar to what was observed by EDS but suggested that the top layer of FR4 probed by XPS was enriched in hydrocarbons (possibly including adventitious carbon) compared to the underlying bulk. In contrast to the EDS result on the Al sample surface, XPS analysis of this surface showed no trace of aluminum; the surface was dominated by C and O, with minor impurities of Si, Cr, Na, Mg, etc., as shown in Table 3. The presence of C and O atoms could be enhanced by contamination of the sample, given that the Alodine chromate conversion treatment of aluminum is generally not performed with high-purity solutions and is likely to leave organic material (as well as the metal and nonmetal impurities) on the surface [29, 30]. XPS analysis of the as-received CG surface revealed atomic concentrations of 24.1% Mg, 50.95% F, 21.81% C, and 3.14% O, thus confirming that the solar cell cover glass had a MgF_2_ coating that was thicker than the probe depth of XPS. The presence of C and O on the CG surface is an indication of adventitious carbon contamination. XPS analysis of the as-received FEP sample surface showed that this material’s surface was composed essentially exclusively of C and F atoms, with atomic percentages of 34% C and 66% F being very close to the theoretical ratio for C:F of 1:2. Indeed, XPS analysis of FEP typically shows FEP surfaces to have C:F atomic concentrations of 1:2 with little contamination [31].

**Table 2:** Elemental compositions of the surface of the as-received FR4 sample, obtained from EDS and XPS analysis

Element	EDS	XPS
C	55.2%	73.5%
O	32.1%	18.3%
Si	8.5%	1.0%
Al	1.1%	0.8%
N	–	2.8%
Impurities (S, Cl, Ca, Ba, Sn)	3.1%	3.6%

**Table 3:** Elemental compositions of the surface of the as-received Al sample, obtained from EDS and XPS analysis

Element	EDS	XPS
C	17.3%	77.2%
O	18.8%	21.4%
Al	58.1%	–
Si	–	1.0%
Zn	1.6%	-
Cr	1.4%	0.2%
Other impurities (Ag, Cu, S, Na, Mg)	2.8%	0.2%

**Fig. 4:**
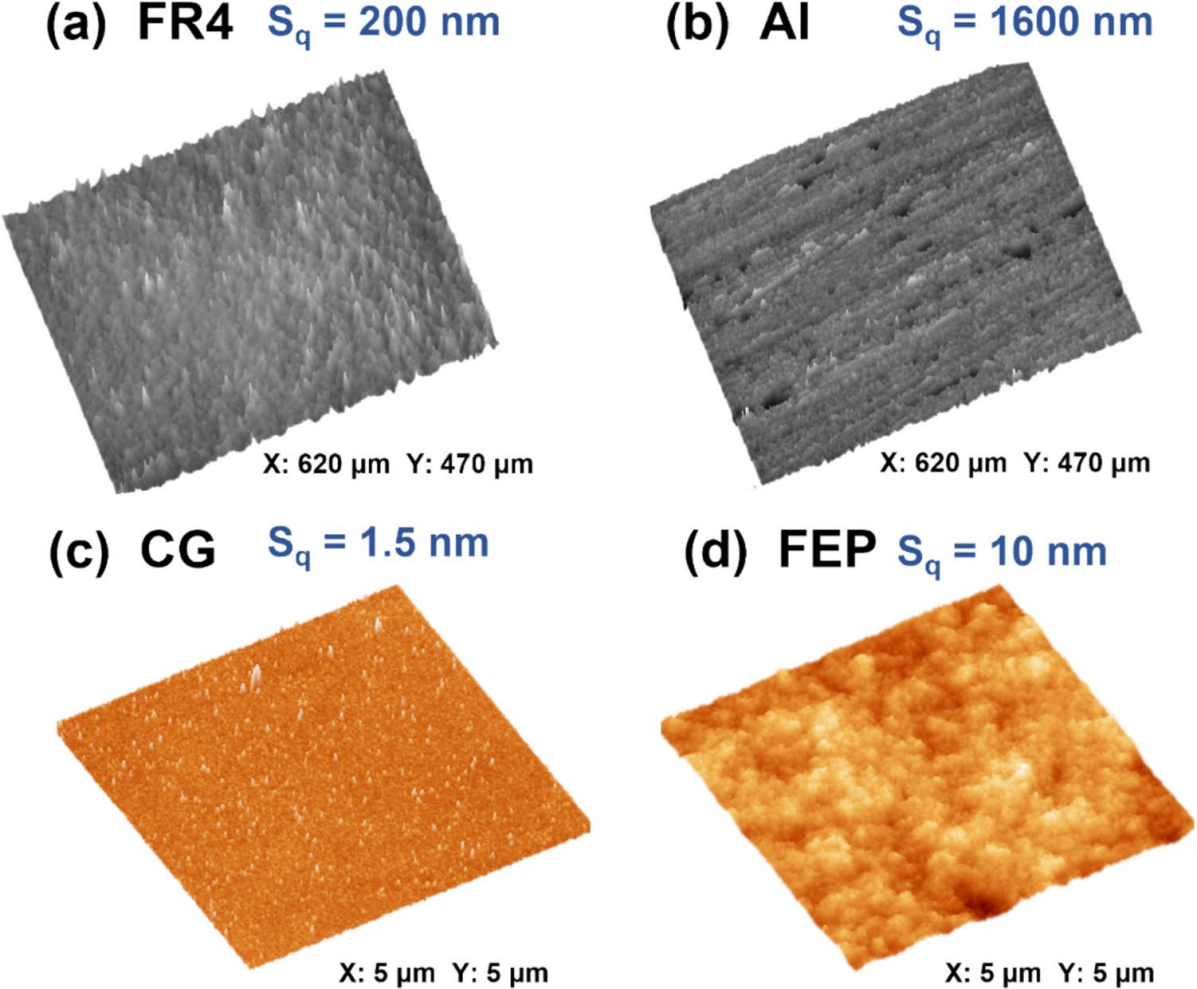
**a**, **b** Representative light interferometric images (620 μm × 470 μm) of FR4 and Al sample surfaces. **c**, **d** Representative AFM images (5 μm × 5 μm) of CG and FEP sample surfaces. The root-mean-square (RMS) surface roughness (Sq) corresponding to each surface is shown

The surface roughnesses of the FR4 and CG samples were quantified by white light interferometry, and those of the CG and FEP samples were quantified by AFM. The very rough surfaces of the FR4 and Al made light interferometry the preferred tool. The images obtained for the FR4 and Al surfaces are shown in Fig. 4a,b. These surfaces were measured to have root mean square (RMS) roughnesses of *S*_*q*_ = 200 and 1600 nm, respectively. The RMS roughnesses derived from AFM images of the CG and FEP samples (Fig. 4c,d) were *S*_*q*_ = 1.5 and 10 nm, respectively. The roughness values obtained from light interferometry and from AFM cannot be compared directly, as the techniques are different and the surface sampling areas are vastly different. Nevertheless, it is clear that the FR4 and Al surfaces are exceedingly rough compared to the CG and FEP surfaces and that the FR4 surface is smoother than the Al surface and the CG surface is smoother than the FEP surface.

Finally, it should be noted that SEM, EDS, and XPS data were collected on the samples after they were exposed to the hyperthermal O/O_2_ beam in the experiments, and no changes in surface morphology or chemical composition were observed. The lack of change in morphology is understandable because the O-atom fluence (< 10^19^ O atoms cm^−2^) on the samples in these molecular beam-surface scattering experiments is sufficiently low that the expected additional roughening of an organic layer on the FR4 and Al surfaces would be less than the roughness of the as-received surfaces and would therefore not be detectable by SEM at the resolution used. Furthermore, relatively unreactive materials, such as MgF_2_ (CG) and FEP, would likely not exhibit any changes in surface roughness with this low AO fluence even at the resolution of an AFM image. The lack of change observed in the EDS or XPS analysis may indicate that little or no reaction occurred, that the small amount of etching resulting from the low-fluence AO exposure largely preserved the chemistry near the surface, and/or that adventitious carbon may have re-contaminated the sample after it was removed from the vacuum chamber. Adventitious carbon likely played a role, because the as-received samples were cleaned in an organic solvent (isopropanol) and passed through a laboratory environment and the exposed samples were examined after they had been stored in a laboratory environment for at least a few weeks.

### Approach to analysis of scattering dynamics data

We investigated the inelastic scattering dynamics of O atoms on the various target surfaces by directing the hyperthermal O/O_2_ beam at the surface, as described in Fig. 1, and detecting the O and O_2_ products that scattered from the surface with the rotatable mass spectrometer detector. In-plane molecular beam-surface scattering experiments were performed on the four surfaces by acquiring TOF distributions with a variety of incident (*θ*_*i*_ = 60, 45, 30, 15, 0°) and final angles *(θ*_*f*_, *ϕ*_*f*_). Although scattering data were collected for the O_2_ molecules in the beam, the focus of this study was on the scattering dynamics of O atoms, and TOF distributions of O_2_ were primarily collected so that the contribution of dissociative ionization of O_2_ to O^+^ could be subtracted from the TOF distributions of O atoms. The O_2_ component of the beam was only 5% of the total incident beam flux (Fig. 1), making the correction for dissociative ionization (11% of the O_2_ TOF signal) nearly negligible. In addition, it was found that the scattering signal from O_2_ was always very small compared to the O-atom signal, so the possibility of O-atom recombination on the surface to form O_2_ was not investigated. The O atoms apparently reacted to some extent with the organic material on the surfaces of the FR4 and Al samples, and while the reaction products were observed, their dynamics were not pursued. Regardless of the possibility of O–O recombination or reaction of O atoms to form other products, the O-atom signals that were obtained after correction for dissociative ionization represent the non-reactive inelastic scattering dynamics of O atoms on the four target surfaces. However, the relative O-atom fluxes from each material surface cannot be compared.

The O-atom TOF distributions were bimodal and were analyzed by assuming the two limiting cases in inelastic gas-surface dynamics: non-thermal impulsive scattering (IS) and thermal desorption (TD) [32–36]. IS atoms scatter from the surface after a few collisions and retain a significant fraction of their pre-collision kinetic energy. IS products are thus described by a relatively narrow TOF distribution that has a maximum at short flight times. In this case, O-atom products retain memory of the incident conditions, and the IS dynamics depend on the incident angle (*θ*_*i*_) and energy (*E*_*i*_) [37–39]. Typical angular distributions of IS atoms are lobular, or quasi-specular, with maximum flux at a final angle far from the surface normal. On the other hand, when the interaction between the incident atom and the surface is strong enough for a substantial amount of energy to be transferred, the incident atom can become trapped in the atom-surface potential well. Such atoms typically reach thermal equilibrium with the surface before desorbing, and if there is no barrier above the atom-surface desorption energy, the atoms desorb in a Maxwell–Boltzmann (MB) velocity distribution characterized by the surface temperature, with a cos*θ*_*f*_ angular distribution of scattered flux about the surface normal. Thus, TD atoms can be identified by the presence of a broad peak in the TOF distribution at longer flight times compared to those of the IS atoms. The IS and TD contributions can be quantified by fitting the slow component of the TOF distribution with a MB translational energy distribution, which can be converted to a number density distribution as a function of time, *N(t)*, and scaled to match the TD component in the TOF distribution. The IS contribution is then calculated by subtracting the TD component from the overall TOF distribution [17, 21, 23]. In general, this analysis is conducted by assuming that the atoms in the incident beam pulse arrive at the sample at the same time, corresponding to the arrival time of atoms with the average of the translational energy distribution of atoms in the incident beam pulse [35]. In cases of grazing scattering, where the average energy transfer in the atom-surface collisions may drop below 40% of the nominal incident energy, this assumption of a monoenergetic beam breaks down, and a more sophisticated analysis procedure must be used, where an assumed translational energy distribution of scattered atoms, *P(E*_*T*_*)*, is adjusted until the convolution of this *P(E*_*T*_*)* distribution and the translational energy distribution of the incident beam, *P(E*_*i*_*)*, gives good agreement with the observed TOF distribution when transformed to a number density distribution as a function of time, *N(t)* [40]. Representative TOF distributions for in-plane scattering (*ϕ*_*f*_ = 0) are shown in Supplementary Information (Figs. SI1, SI3, SI5, and SI7), and the corresponding translational energy distributions, *P(E*_*T*_*)*, that were derived from these TOF distributions are shown in Figs. SI2, SI4, SI6, and SI8. Relative angular flux distributions are computed by integrating the *P(E*_*T*_*)* distributions, as the *P(E*_*T*_*)* distributions are proportional to flux, whereas the TOF distributions are proportional to number density. By default, the total relative flux that includes both IS and TD O atoms is presented in the results below; however, where noted, the IS and TD components are analyzed separately. As may be observed in Figs. SI1-SI8, the signal-to-noise ratios in the TOF distributions and in the *P(E*_*T*_*)* distributions that are derived from them are high; therefore, the integrated fluxes presented below have statistical counting errors that are estimated to be less than ± 5% (0.95 confidence limit).

### Inelastic scattering dynamics of O atoms

**Fig. 5:**
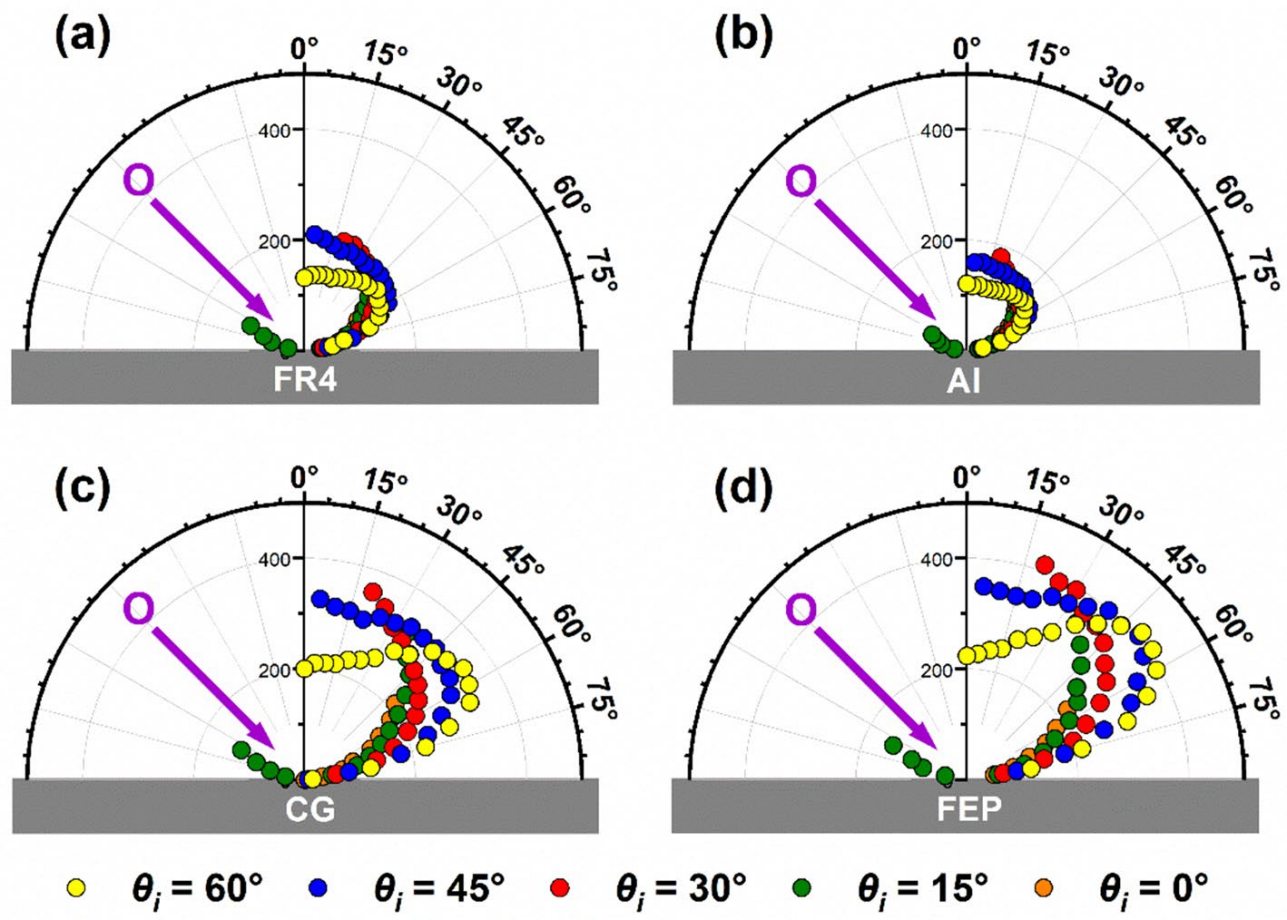
Angular distributions of scattered O-atom flux on the four different surfaces, with the five incident angles used (indicated in the legend underneath the plots), following impingement of O atoms at 〈*E*_*i*_〉 = 451.9 kJ mol^−1^. The flux values are relative and have been normalized so that they may be compared directly across all plots

Figure 5 shows in-plane angular flux distributions for O atoms scattering from the four surfaces with five angles of incidence. While the angular distributions in Fig. 5 show total flux values, the IS and TD fluxes have been separated in the representative distributions for *θ*_*i*_ = 60° and 15° in Figs. SI9 and SI10, respectively. As may be seen from Fig. 5, the angular distributions are clearly quasi-specular when *θ*_*i*_ = 60°, and they become broader and tend more toward cos*θ*_*f*_ when the incident angle decreases. The highest O-atom flux was observed from the FEP surface, followed by CG, FR4, and Al, for all incident angles. Additionally, for *θ*_*i*_ = 60°, the relative O-atom flux from all surfaces increases monotonically from *θ*_*f*_ = 0°, until *θ*_*f*_ ~ 50°and rapidly decreases at higher angles (see Fig. SI9 also). This trend also applies to the data for CG and FEP with *θ*_*i*_ = 45°. On the other hand, for FR4 and Al with *θ*_*i*_ = 45° and all sample surfaces with *θ*_*i*_ = 0, 15, and 30°, the angular distributions exhibit the maximum flux at the closest detectable angle to the surface normal (see Fig. SI10 also). For *θ*_*i*_ = 15°, scattered O atoms were able to be collected in both the forward and backward hemispheres (on the opposite and same sides, respectively, of the surface normal as the incident beam). This result and the observation that the O-atom flux is well above zero at the minimum final angle where products were detected with the larger incident angles suggest that O atoms may generally scatter into both the forward and backward hemispheres.

**Fig. 6:**
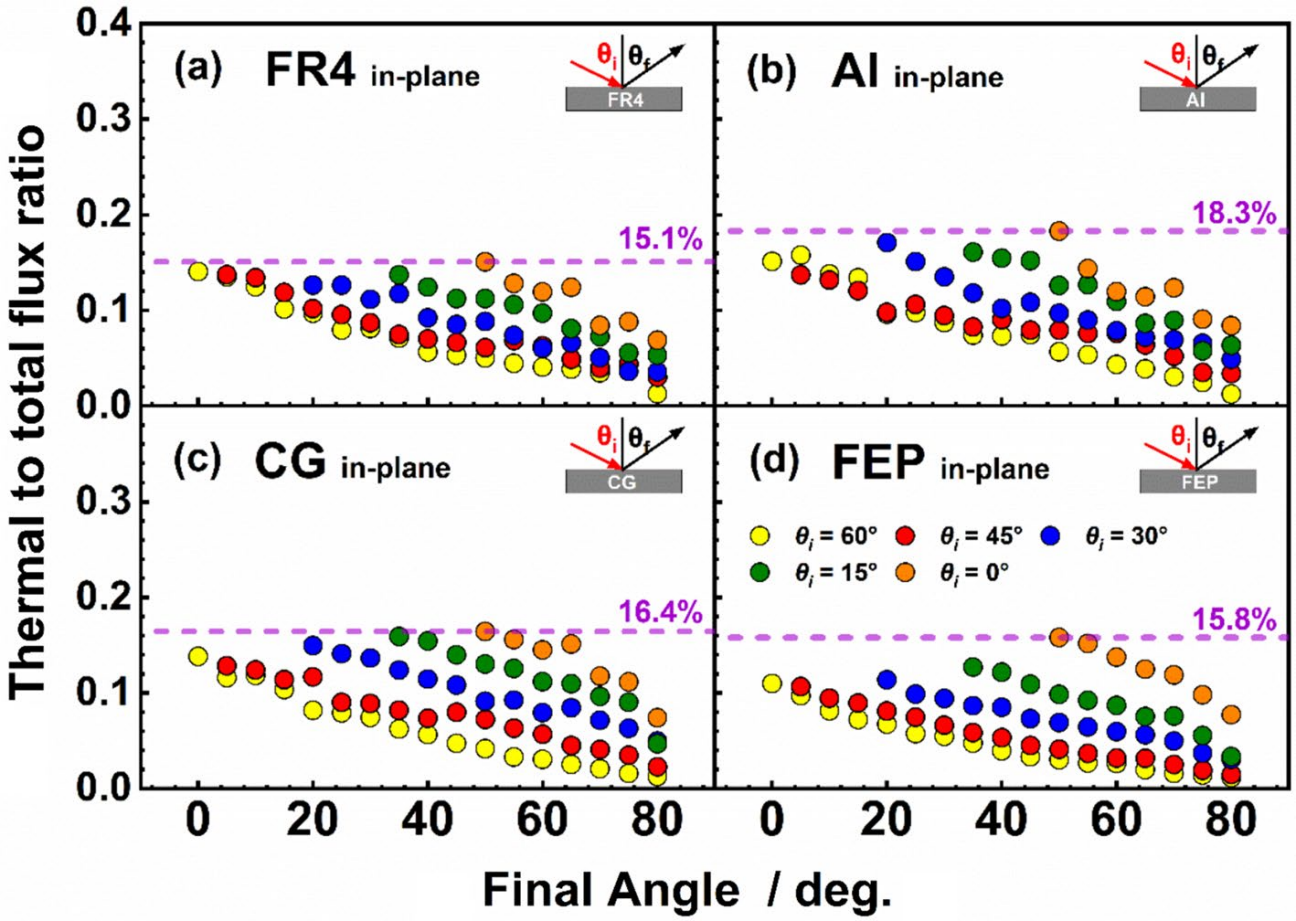
Fraction of TD flux as a function of final angle (*θ*_*f*_) for in-plane scattering of O atoms on **a** FR4, **b** Al, **c** CG, **d** FEP surfaces at the indicated incident angles

Figure 6 shows the fraction of TD flux as a function of *θ*_*f*_ for in-plane inelastic scattering of O atoms on all four surfaces. As may be seen, impulsive scattering dominates, and the TD flux becomes vanishingly small for the most grazing collisions. The TD fraction is somewhat larger for the rougher FR4 and Al surfaces but not remarkably so. Apparently, scattering of hyperthermal O atoms from these disparate surfaces generally proceeds through non-thermal processes, although the energy transfer may be higher on the rougher surfaces (see below).

**Fig. 7:**
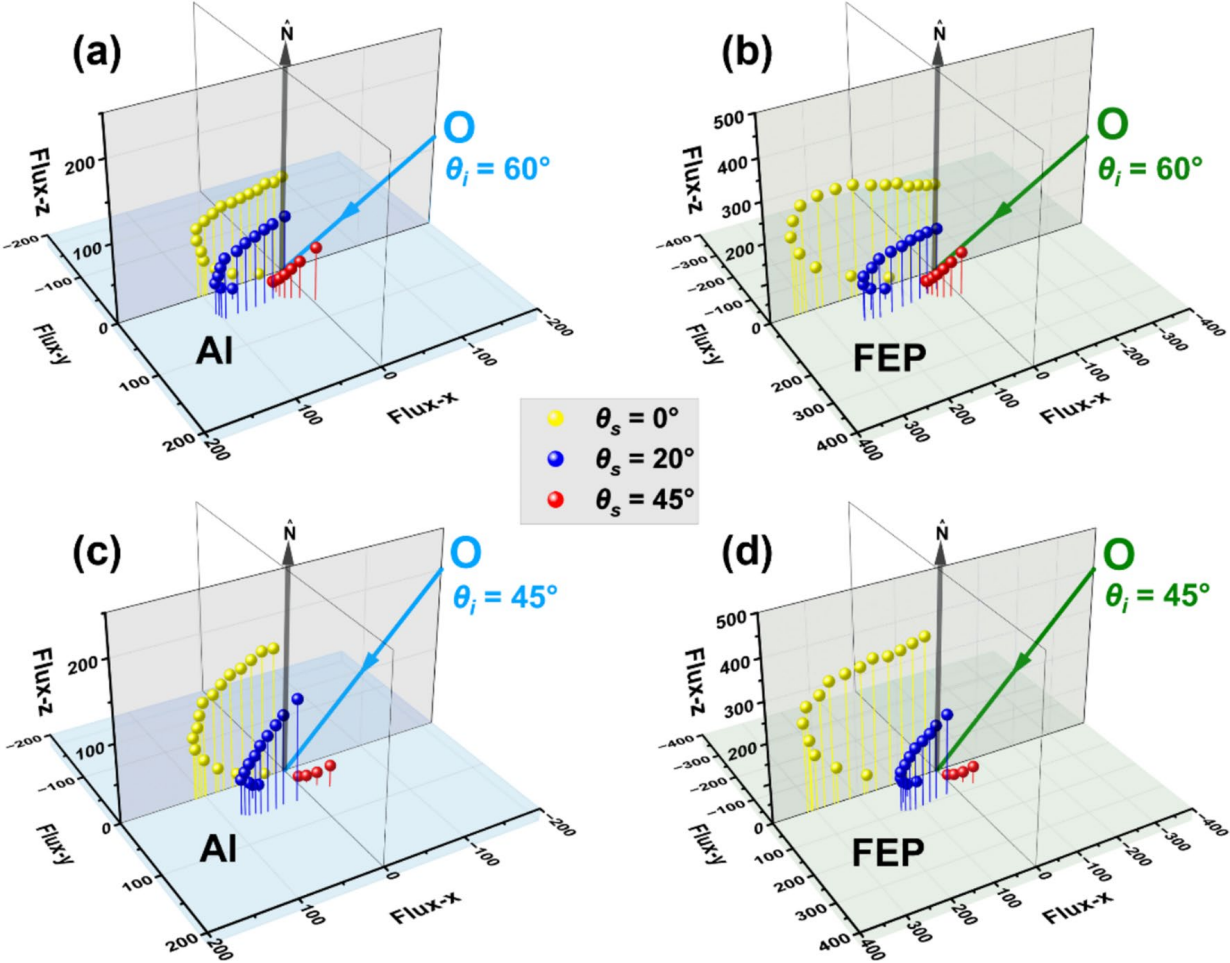
Angular distributions of scattered O-atom flux from Al (**a**, **c**) and FEP (**b**, **d**) with incident angles of 60° (**a**, **b**) and 45° (**b**, **d**). Data shown correspond to sample tilt angles (*θ*_*s*_) of 0° (yellow), 20° (blue), and 45° (red)

Angular flux distributions for in-plane and out-of-plane scattering of O atoms from Al and FEP are shown in Fig. 7 for *θ*_*i*_ = 60 and 45°, and in Fig. SI11 for *θ*_*i*_ = 30° (FEP only). These results show that the O-atom flux at the detected out-of-plane final angles is lower than the detected in-plane flux (for a given *θ*_*f*_). In addition, the O atoms with *θ*_*i*_ = 60° are scattered more forward (i.e., in the direction with larger final polar angles and smaller azimuthal angles) than the O atoms with *θ*_*i*_ = 45° (and *θ*_*i*_ = 30° for FEP). In addition, the out-of-plane angular flux distributions are broader than the in-plane distributions. And for scattering at the largest azimuthal angles (red symbols), the scattered flux increases with *ϕ*_*f*_. This increase is a consequence of *ϕ*_*f*_ being correlated with *θ*_*f*_ in our experiments. If *θ*_*f*_ could be controlled independently of *ϕ*_*f*_*,* then for a given *θ*_*f*_, the flux would be expected to drop with increasing *ϕ*_*f*_.

The energy transfer of the O atoms that scatter from the surface is characterized by the average transfer of translational energy to the surface as a fraction of the incident energy (where the thermal energy of the gas atom at the surface temperature (2*k*_*B*_*T* ≈ 0.062 kJ mol^−1^ at 373 K is taken to be negligible compared to the incident energy):

4 $$\frac{{\left\langle {E_{i} } \right\rangle \; - \;\left\langle {E_{f} } \right\rangle }}{{\left\langle {E_{i} } \right\rangle }}\;\; = \;\;\frac{{\left\langle {\Delta E} \right\rangle }}{{\left\langle {E_{i} } \right\rangle }}$$

**Fig. 8:**
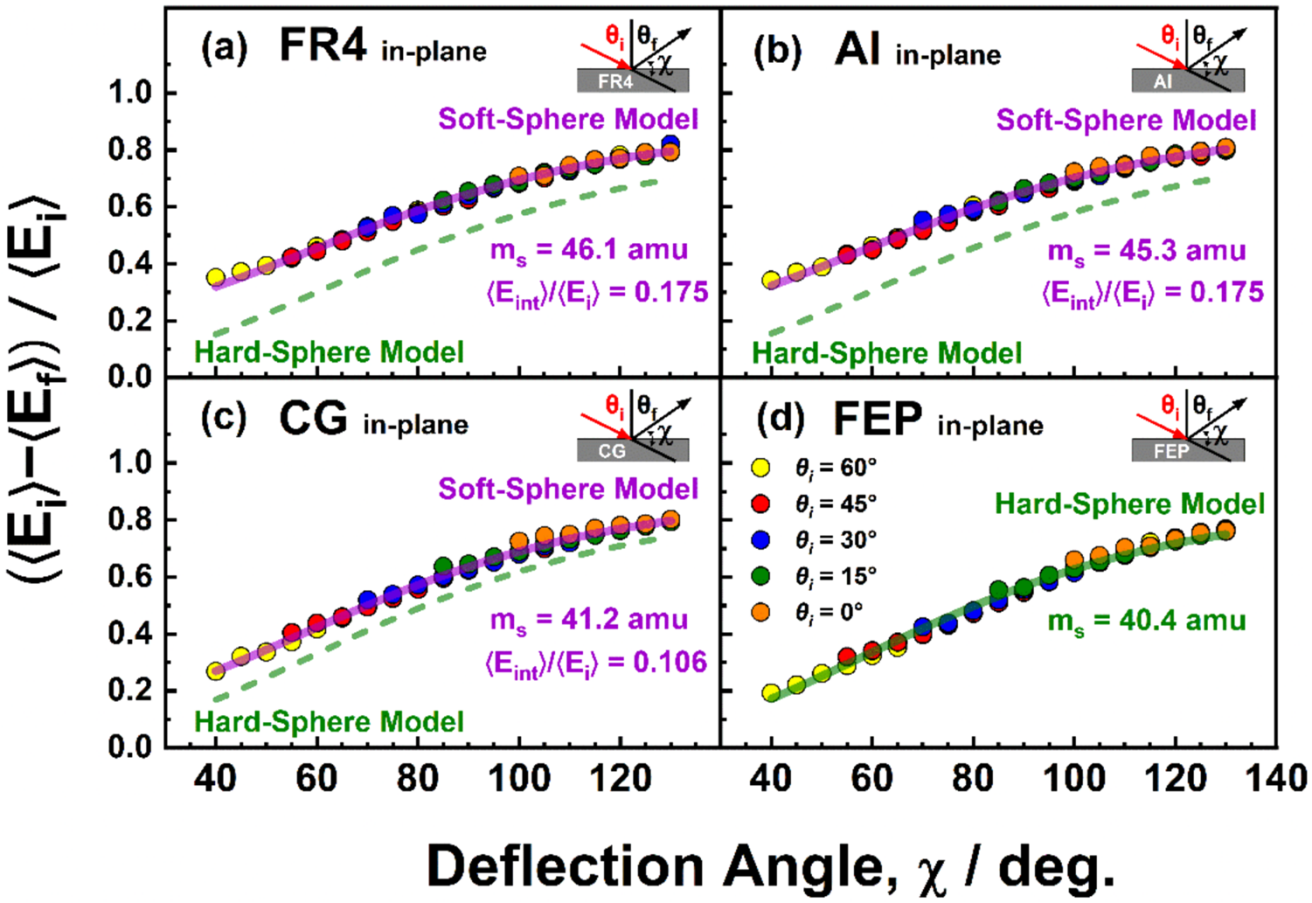
Average fractional energy transfer for in-plane scattering of O atoms from FR4, Al, CG, and FEP as a function of deflection angle, χ, defined as χ = 180° − (*θ*_*i*_ + *θ*_*f*_), where *θ*_*i*_ and *θ*_*f*_ are the incident and final (scattering) angles, respectively. The curve through the data is predicted by a soft-sphere (violet curve) [32] or hard-sphere (green curve) [43] model, with effective surface masses and internal energy excitations (in the center-of-mass frame) shown. Estimated errors are ± 2% for χ > 60° and grow to ± 5% for χ = 40° (0.95 confidence limit)

The average fractional energy transfers for in-plane scattering of O atoms from the four surfaces studied corresponding to each pair of incident and final angles is represented in Fig. 8, where 〈Δ*E*〉/〈*E*_*i*_〉 is plotted as a function of the deflection angle, χ, which is the angle through which the incident atoms are deflected when they scatter from the surface. In other words, the deflection angle is the angle between the direction in which the atom exits the surface and the direction of propagation of the atom if the surface had not deflected it. Thus, for in-plane scattering, χ = 180° − (*θ*_*i*_ + *θ*_*f*_), where the sum, *θ*_*i*_ + *θ*_*f*_, is the angle between the atom’s incident and final directions. The results in Fig. 8 demonstrate that the energy transfer depends on the deflection angle and not on the incident or final angle alone.

The scattering dynamics represented in Figs. 5, 7, and 8 are indicative of scattering in the structure regime [41, 42]. In this regime, incident atoms scatter from a surface that is rough on the atomic scale, and the momentum of the incident atom parallel to the surface is not conserved. When such atom-surface collisions are impulsive, energy transfer has been described by sphere-sphere scattering models, and in Fig. 8, the data are fit well for FR4, Al, and CG by a soft-sphere model [32] and for FEP by a hard-sphere model [43], with effective surface masses of about 40–46 amu. Note that the hard-sphere model curves with the same effective surface mass found for the soft-sphere model fits are shown for comparison in Fig. 8a-c. Transfers of incident translational energy to internal excitation of the surface in the center-of-mass frame are represented by the average internal energy excitation as a fraction of the average incident energy 〈*E*_int_〉/〈*E*_*i*_〉, and these fractions are 0.175 for FR4 and Al, 0.106 for CG, and 0 for FEP. In the structure scattering regime, angular flux distributions of atoms that scatter impulsively on a rough surface commonly show quasi-specular scattering for grazing-incidence collisions, with the cos*θ*_*f*_ character of the angular distributions increasing for more normal incidence or increased surface roughness [44]. Both situations enhance scattering angle randomization resulting from multiple-bounce trajectories on the surface. In addition, incident angles closer to normal lead to more “head-on” collisions with the local surface moieties involved in the collision and therefore more energy transfer and more loss of parallel momentum.

The relationship between average fractional energy transfer and deflection angle may be extended to include out-of-plane scattering, where both the polar and azimuthal angles change as a result of the atom-surface collision. The deflection angle is still the difference between 180° and the angle between the scattering atom’s incident and final directions. In terms of *θ*_*i*_*, θ*_*f*_, and *ϕ*_*f*_, the deflection angle is given by [45]:

5 $${\upchi } = {\mathrm{arccos}}\left( { - \cos \theta_{i} \cos \theta_{f} - \sin \theta_{i} \sin \theta_{f} \cos \left( {180^\circ - \phi_{f} } \right)} \right)$$

**Fig. 9:**
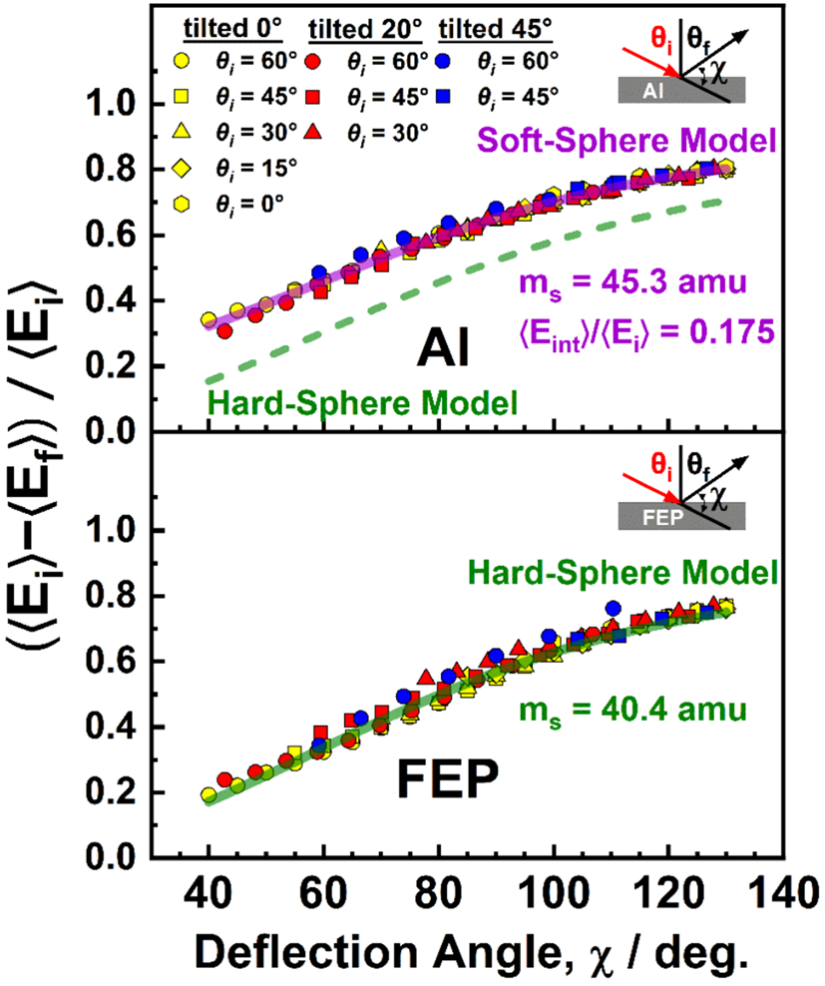
Average fractional energy transfer of O atoms that scatter from Al and FEP as a function of deflection angle, χ, defined in Eq. (5), where *θ*_*i*_ is the incident angle, and the tilt angle of the sample, *θ*_*s*_, is indicated (see Fig. 2). The curves through the data are predicted by (top panel) a soft-sphere model [32] with an effective surface mass of 45.3 amu and a transfer of 17.5% of the incident energy in the c.m. reference frame to internal modes of the surface and (bottom panel) a hard-sphere model [43] with an effective surface mass of 40.4 amu and a transfer of 0% of the incident energy in the c.m. reference frame to internal modes of the surface. Estimated errors are ± 2% for χ > 60° and grow to ± 5% for χ = 40° (0.95 confidence limit)

Figure 9 shows plots of average fractional energy transfer for all incident and all final angles where data were collected for both in-plane and out-of-plane O-atom scattering from Al and FEP. As is evident by the alignment of all the energy transfer data along curves that match either the soft-sphere or hard-sphere model fits, the dependence of average fractional energy transfer on deflection angle is remarkably robust.

## Discussion

The angular distributions of scattered O-atom flux in Figs. 5 and 7 suggest that the flux of O atoms from FR4 and Al is lower than that scattered from CG and FEP. While out-of-plane scattering (including scattering into the unexplored backward hemisphere) likely plays a role in the apparent differences in scattered flux, the reactivity of the surfaces to O atoms is undoubtedly the main reason for these differences. The EDS and XPS results in Tables 2 and 3 revealed significant carbon on the FR4 and Al surfaces, which was presumably in the form of organic hydrocarbons. The reaction of O atoms with these surfaces was verified by the observation of OH products. As inferred from an earlier study in our laboratory on the scattering of hyperthermal O atoms on a liquid hydrocarbon surface [17], the reaction probability to form OH may be ~ 1/2 that for an incident O atom to scatter without reacting. O atoms might also react to form H_2_O, which would further reduce the fraction of O atoms that scatter from the surface without reacting. There was evidently some adventitious carbon (again presumed to be in the form of hydrocarbons) on the CG surface, although it is unclear what the extent of adventitious carbon contamination on the CG surface was during the scattering experiments, where the surface was warmed in vacuum and bombarded by O atoms. It is possible that this surface was slightly reactive to incident O atoms, but this reactivity was not confirmed. Earlier work in our laboratory showed that O atoms with incident energies greater than ~ 386 kJ mol^−1^ can react directly with FEP to produce CFO and CF_2_O products; however, these signals were weak, and the incident energy used for the present experiments, 451.9 kJ mol^−1^, is not far above the threshold energy, so the reactivity of FEP with O atoms in these experiments is expected to be much less than the reactivity with the hydrocarbon-covered surfaces of FR4 and Al and possibly less than the hydrocarbon-contaminated surface of CG. Thus, it is not surprising that the scattered O-atom flux from FEP is much higher than that from FR4 and Al and slightly higher than that from CG. Nevertheless, regardless of the magnitudes of their relative flux, the dynamical behavior of the detected O atoms that scatter from all four surfaces may be assumed to be representative of the non-reactive scattering dynamics on these realistic satellite materials.

The data presented in Figs. 5, 7, 8 and 9 (and in Fig. SI11) pertain to the sum of all O atoms that scatter at a particular set of incident and final angles; nevertheless, the analysis procedure typically assumes that non-thermal (IS) and thermal desorption (TD) scattering events may be separated into two distinct components (see Figs. SI1-SI10). As there is no analytical form to fit impulsive scattering, information associated with the IS component is obtained by fitting a MB distribution to the TOF distribution and subtracting it from the overall TOF distribution. After the TD component has been subtracted, the resultant IS contribution exhibits a slow tail in the TOF distribution that corresponds to a low-energy peak in the translational energy distribution. The IS translational energy distributions thus contain two components (Figs. SI2, SI4, SI6, SI8). Earlier work in our lab [40] has shown that the dominant component, with higher final energies, corresponds to a lobular – i.e., non-cos*θ*_*f*_ – angular flux distribution, whereas the lower-energy component corresponds to atoms that scatter with a roughly cos*θ*_*f*_ distribution, albeit with slightly non-thermal final energies. The conclusion of the earlier work was that the lower-energy IS component, with a cos*θ*_*f*_ angular flux distribution, was the result of scattering angle randomization from trajectories with multiple inner turning points (“multiple bounces”). This phenomenon of scattering angle randomization was investigated further in a subsequent study that described the impulsive scattering of O atoms on liquid surfaces in terms of cosine and non-cosine character [44]. Thus, even on a liquid surface, which has only local corrugation from the molecular chains of the liquid and their thermal motions (without geometrical roughness akin to that of the four surfaces studied here), hyperthermal incident O atoms can apparently scatter over a broad range of angles and lose significant energy as a result of multiple-bounce collision trajectories.

The overall energy transfer to the surface may be strongly affected by the fraction of incident atoms that exit the surface via thermal desorption, as TD implies complete accommodation of the incident atom’s energy on the surface. Potential well depths are far lower than the incident O-atom energies used in this work. Therefore, atoms must lose a substantial amount of their incident energy to the surface before becoming trapped by the potential well. Thermal accommodation may occur through a single inner turning point event, but it is more likely to occur through sequential loss of energy through multiple bounces on the surface. Figure 6 depicts the fraction of TD as a function of final angle for in-plane scattering of O atoms from the four surfaces studied at the various incident angles used. These plots show that, for all surfaces, the fraction of TD atoms increases for low *θ*_*f*_ angles (high deflection angles), where energy transfer is greater (see Fig. 8). The fraction of TD atoms may have a weak correlation with the nature of the surface, but it is not possible to determine whether the TD fraction is controlled more by the atom-surface potential well or other parameters that characterize surface roughness. The plots in Fig. 6 show a pronounced similarity in the dependence of TD fraction on incident and final angles for all the surfaces investigated. In addition, the TD fractions are relatively small, decreasing from 15–18% for more incident and final angles closer to normal to only a few percent for grazing scattering trajectories.

The shapes of the angular flux distributions are similar regardless of the measured surface roughness (Figs. 5 and 7). The most grazing incident angle (*θ*_*i*_ = 60°) results in quasi-specular scattering with a maximum flux slightly less than the specular angle, and decreasing incident angles lead to angular flux distributions that become proportionally broader for scattering on all surfaces. Close inspection of the angular distributions reveals noticeably broader angular flux distributions from the FR4 and Al surfaces, but they are surprisingly similar to those from the CG and FEP surfaces given the differences in RMS surface roughness that span nearly three orders of magnitude.

The fact that the angular distributions are broad, both in-plane and out-of-plane, might be interpreted as the result of atom-surface scattering trajectories with multiple inner turning points. Such trajectories could occur on a surface that has geometrical roughness, with peaks and valleys on the surface having dimensions that are large compared to atomic dimensions. In addition, a corrugated atom-surface interaction potential arising from atomic roughness in the localized region of the atom-surface collision might lead to trajectories with multiple inner turning points. Such multiple-bounce trajectories would indeed be expected to randomize the scattering angles. The generally higher fraction of out-of-plane vs. in-plane O-atom flux from Al in comparison with that from FEP is consistent with the supposition of more multiple-bounce trajectories from the geometrically rougher Al surface. In addition, the average fractional energy transfers on the Al surface tend to be slightly higher than those for the FEP surface (Fig. 9), again suggesting a higher fraction of multiple-bounce scattering events on the rougher Al surface, which lead to an increase in energy transfer. On the other hand, the fractional energy transfers from the FR4 surface are essentially identical to those from the Al surface (Fig. 8a,b), even though the FR4 surface is apparently much smoother (*S*_*q*_ = 200 nm for FR4 vs. 1600 nm for Al). However, the energy transfers on the CG surface are somewhat higher than those on the FEP surface even though the CG surface is smoother (*S*_*q*_ = 1.5 nm for CG vs. 10 nm for FEP).

The idea that multiple-bounce trajectories are the dominant effect on the scattering dynamics might also be difficult to reconcile with the fact that the dependence of average fractional energy transfer on the deflection angle is described nearly perfectly by a sphere-sphere scattering model that assumes a single collision between the incident atom and the surface (i.e., a single inner turning point in the trajectory). However, our earlier theoretical study of Ar scattering on a self-assembled monolayer surface indicated that collisions leading to non-thermal scattering with multiple inner turning points correlate with approximately the same effective surface mass as collisions with a single inner turning point [32], which, in turn, suggests that the sphere-sphere scattering model may be applicable to situations where multiple-bounce scattering events dominate, even though this is non-intuitive. Furthermore, the earlier theoretical result points to the possibility that the applicability of the sphere-sphere model may be more robust for hyperthermal incident energies (i.e., those used for the present experiments) because an atom could undergo many inner turning points in its trajectory and still scatter before reaching thermal equilibrium. Although the interplay between the localized atom-surface interaction potential and the geometrical surface roughness undoubtedly has a quantitative effect on the atom-surface scattering dynamics, the striking similarity in the O-atom scattering dynamics on the four disparate surfaces investigated suggests that these details do not dominate the scattering dynamics. We speculate here that the scattering dynamics on these four surfaces are largely governed by the nature of the incident atom and its high incident energy. Further study is warranted to uncover the factors that control atom-surface scattering dynamics under various scenarios.

The energy transfer function and the angular flux distributions for a variety of incident angles and both in-plane and out-of-plane final angles provide detailed information on the scattering dynamics that may be used in the development of a gas-surface scattering model. Such a model may then be used to calculate the overall energy and momentum transfer for inelastic scattering of incident atoms under a given set of conditions. When considering the O-atom scattering dynamics on CG and FEP as compared to FR4 and Al, Fig. 5 shows that the CG and FEP surfaces promote O-atom scattering over a narrower angular range and with lower average deflection angles than exhibited by the angular flux distributions collected from the FR4 and Al surfaces. In addition, the fractional energy transfer functions for CG and FEP indicate only slightly lower energy transfers than the functions for FR4 and Al, which are essentially identical to each other. Thus, the difference in the overall energy (or momentum) accommodation of O atoms on CG and FEP vs. FR4 and Al is expected to be mediated mainly by the angular flux distributions, resulting in much lower energy accommodation coefficients on CG and FEP than on FR4 and Al. The out-of-plane O-atom flux is likely to be significant, as suggested by the data on the Al and FEP surfaces in Fig. 7, and knowing the angular distribution of this flux will therefore be imperative to quantify the energy accommodation coefficient, even if the fractional energy transfer function can be deduced from in-plane scattering measurements (which appears to be the case from the data in Fig. 9). An accurate gas-surface model that could be parametrized with both in-plane and out-of-plane scattering data could be used to calculate the energy accommodation coefficient. However, it would be impractical to collect detailed scattering dynamics data to parametrize such a gas-surface scattering model for every potential case of incident projectile and type of surface. Based on the similarities in the scattering dynamics for hyperthermal O atoms scattering on the four different surfaces investigated in this study, we postulate that, by collecting such data for a sufficient number of model systems, rules of thumb could be developed for predicting model parameters from knowledge of the chemical nature of the material and measurements of the surface properties, for example, details pertaining to surface roughness.

## Conclusion

The scattering dynamics of hyperthermal O atoms on four representative satellite materials (FR4 − circuit board material; Al – aluminum with an Alodine chromate conversion coating; CG – solar cell cover glass with a MgF_2_ coating; FEP – fluorinated ethylene propylene polymer) were investigated with a molecular beam-surface scattering technique. Pulsed beams of ~ 7.5 km s^−1^ O atoms were directed at the target surfaces, and the non-reactively scattered O atoms were detected by a rotatable mass spectrometer as a function of incident and final polar angles, *θ*_*i*_ and *θ*_*f*_, respectively, and final azimuthal angle, *ϕ*_*f*_, as well as final translational energy, *E*_*T*_. The translational energy distributions revealed both impulsive scattering (IS) and thermal desorption (TD). The IS component was bimodal and corresponded to O atoms that exited the surface nonthermally in a quasi-specular non-cos*θ*_*f*_ angular flux distribution and a cos*θ*_*f*_ distribution, as observed in earlier experiments with hyperthermal O atoms scattering on liquid surfaces [40, 44]. The quasi-specular IS component was overwhelmingly dominant over the cosine IS component, and it was also dominant over the TD scattering pathways. Nevertheless, the TD O atoms were not negligible and accounted for a fraction of O-atom scattering events that ranged from a few percent to ~ 18%.

Surface characterization of the target materials showed that the CG and FEP surfaces were relatively smooth compared to the FR4 and Al surfaces. Moreover, chemical composition analysis showed high concentrations of carbon and oxygen on the as-received FR4 and Al surfaces, suggesting that O atoms incident on these surfaces interacted with a hydrocarbon layer. Higher-fluence AO exposures of these materials (e.g., in VLEO) would be expected to remove the < 1 μm-thick hydrocarbon layer on the Al surface, leading to O-atom scattering on what would likely become an unreactive oxygen-covered surface of oxidized aluminum. Nevertheless, the scattering dynamics of O atoms on this surface are probably dominated by the geometrical roughness, and the scattering dynamics would probably not change qualitatively even if the hydrocarbon layer were removed. Unlike the Al sample, FR4 is a composite material of glass fibers in an epoxy matrix; thus, the hydrocarbon phase would continue to ablate with longer AO exposures, creating a complex and very rough surface with unreacted fibers and deep etched regions in between. The geometrical roughness would be significantly enhanced, and it is difficult to predict from the current data how the scattering dynamics would change.

The observed angular distributions of scattered O-atom flux were qualitatively similar from all surfaces, but they were quantitatively broader from the FR4 and Al surfaces than from the CG and FEP surfaces. This observation is consistent with the significantly higher RMS roughness of the FR4 and Al surfaces, which can lead to multiple-bounce scattering trajectories of the O atoms as they interact with the surface. Additionally, the scattered O-atom flux was lower from the FR4 and Al surfaces than from the CG and FEP surfaces. The lower flux was attributed mainly to reactive scattering of O atoms with the hydrocarbons on these surfaces to produce OH, H_2_O, and perhaps other products.

The average fractional energy transfers between the incident O atoms and the tested surfaces were found to depend on the deflection angle, and the energy transfer functions were fit well by a sphere-sphere scattering model, as has been observed in many prior studies [32, 40, 42]. The average energy transfers were slightly higher on the FR4 and Al surfaces than on the CG and FEP surfaces. Nevertheless, the energy transfer functions for all four surfaces indicated similar energy transfers.

Based on the scattering dynamics that have been revealed through angular flux distributions and energy transfer functions, we expect that the FR4 and Al surfaces would promote greater overall energy accommodation of incident hyperthermal O atoms than would the CG and FEP surfaces. Obtaining quantitative results for the total incident particle translational energy or momentum accommodation to the surface will require reliable gas-surface interaction models to extrapolate the angular flux distributions and energy transfer functions obtained experimentally to all final scattering angles.

## Supplementary Information

Below is the link to the electronic supplementary material.

Supplementary file1 (PDF 1562 KB)

## Data Availability

Numerical data is available upon request.
