# Supplementary material for: Inelastic scattering dynamics of hyperthermal O atoms on engineering surfaces relevant to satellites in low Earth orbit
Source: CEAS Space J. 2025 Jun 27;18(3):541–58. doi: 10.1007/s12567-025-00628-5 (PMC13144222; doi:10.1007/s12567-025-00628-5)
Supplement: Supplementary file 1 — Supplementary file1 (PDF 1562 KB) [file 12567_2025_628_MOESM1_ESM.docx]

***Supplementary information***

***for***

**Inelastic Scattering Dynamics of Hyperthermal O Atoms on Engineering Surfaces Relevant to Satellites in Low Earth Orbit**

**Chenbiao Xu****^^[[1]](#footnote-2)^,^**^†^ **· Adriana Caracciolo^1^ · Pedro D. C. Jorge^1^ · Irina Gouzman^[[2]](#footnote-3),^**^‡^ **· Marcin D. Pilinski^[[3]](#footnote-4)^ · and Timothy K. Minton^1,^***

**Fig. SI2** Translational energy distributions for in-plane O-atom scattering from CG with an average incident energy of 〈*E_i_*〉 = 451.9 kJ mol^-1^, derived from the TOF distributions in Fig. SI1. The experimental data are shown as yellow symbols, and the corresponding incident and final angles, *θ_i_* and *θ_f_*, respectively, are indicated in each panel. The red curves are MB fits to the thermal desorption TD components, and the differences between the experimental TOF distributions and the MB fits are taken to be the IS components (blue curves). The TD flux is relatively low, and the integrals of the TD components are almost negligible compared to those of the IS components.


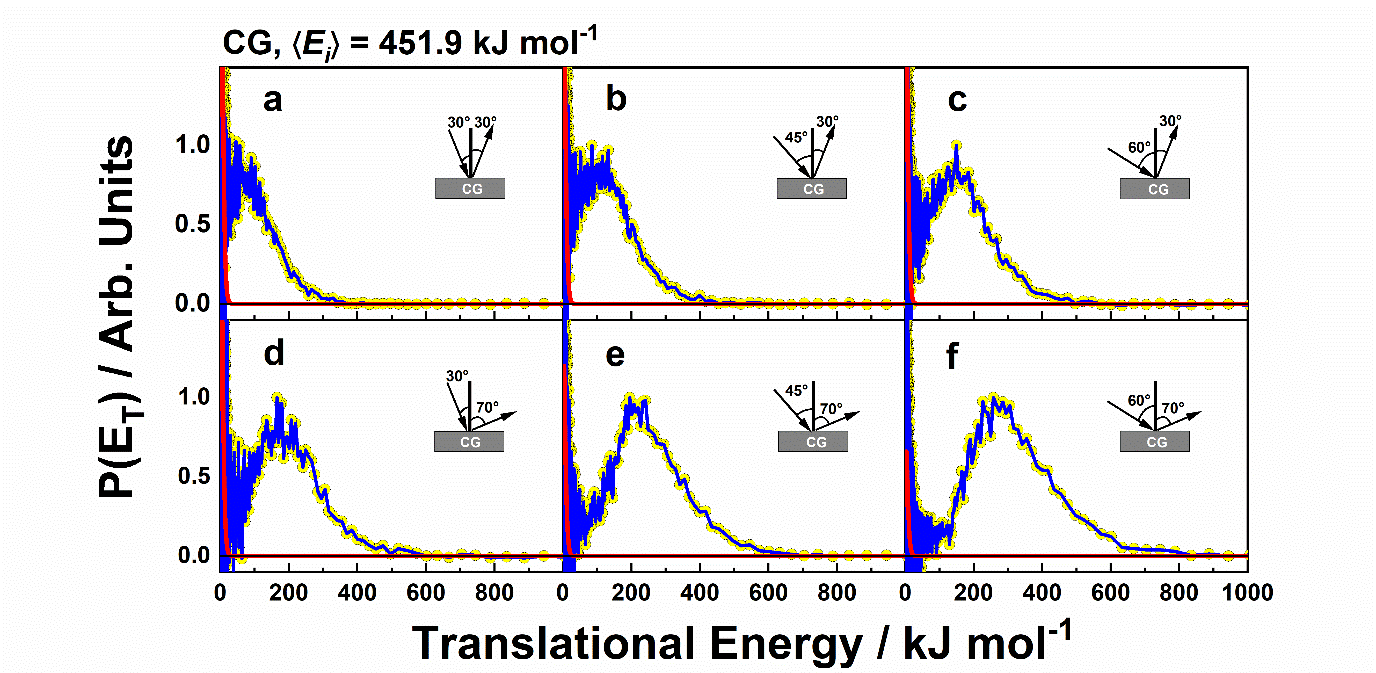


**Fig. SI1** Representative time-of-flight distributions for in-plane O-atom scattering from CG with an average incident energy of 〈*E_i_*〉 = 451.9 kJ mol^-1^. The experimental data are shown as yellow symbols, and the corresponding incident and final angles, *θ_i_* and *θ_f_*, respectively, are indicated in each panel. The red curves are Maxwell-Boltzmann (MB) fits to the thermal desorption (TD) components, and the differences between the experimental TOF distributions and the MB fits are taken to be the IS components (blue curves).


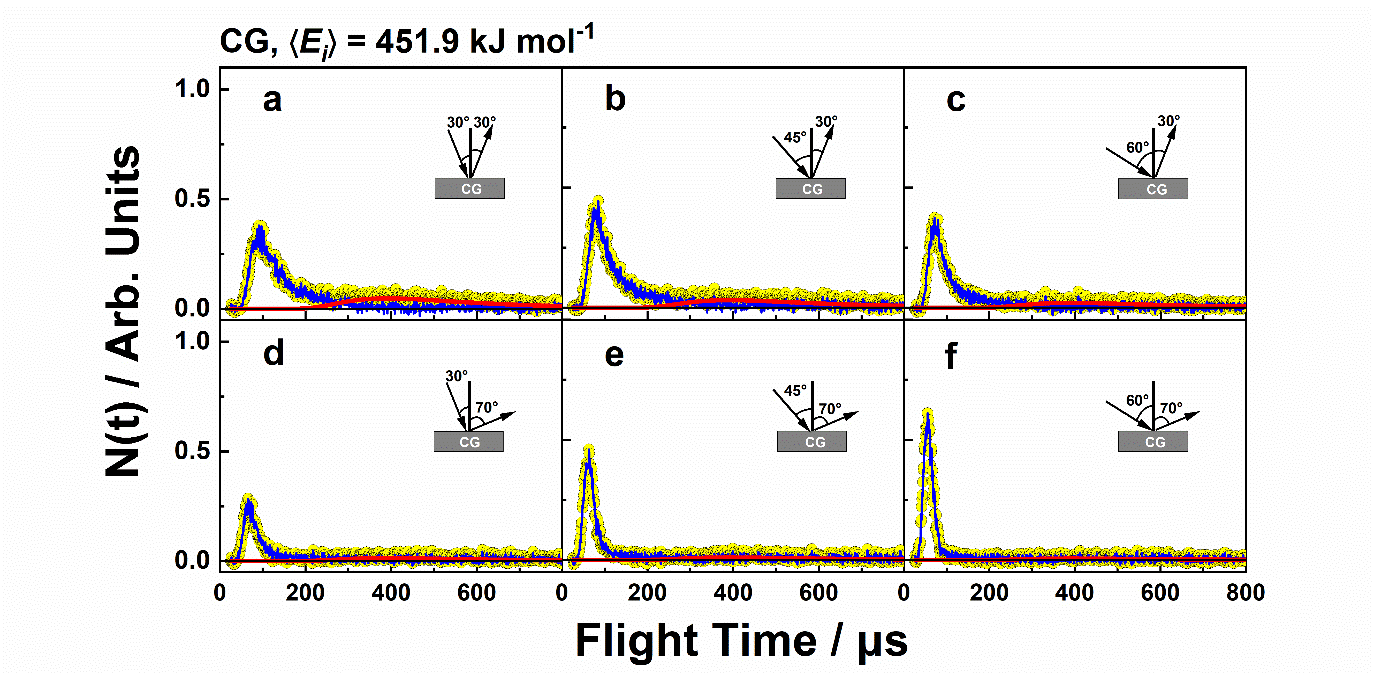

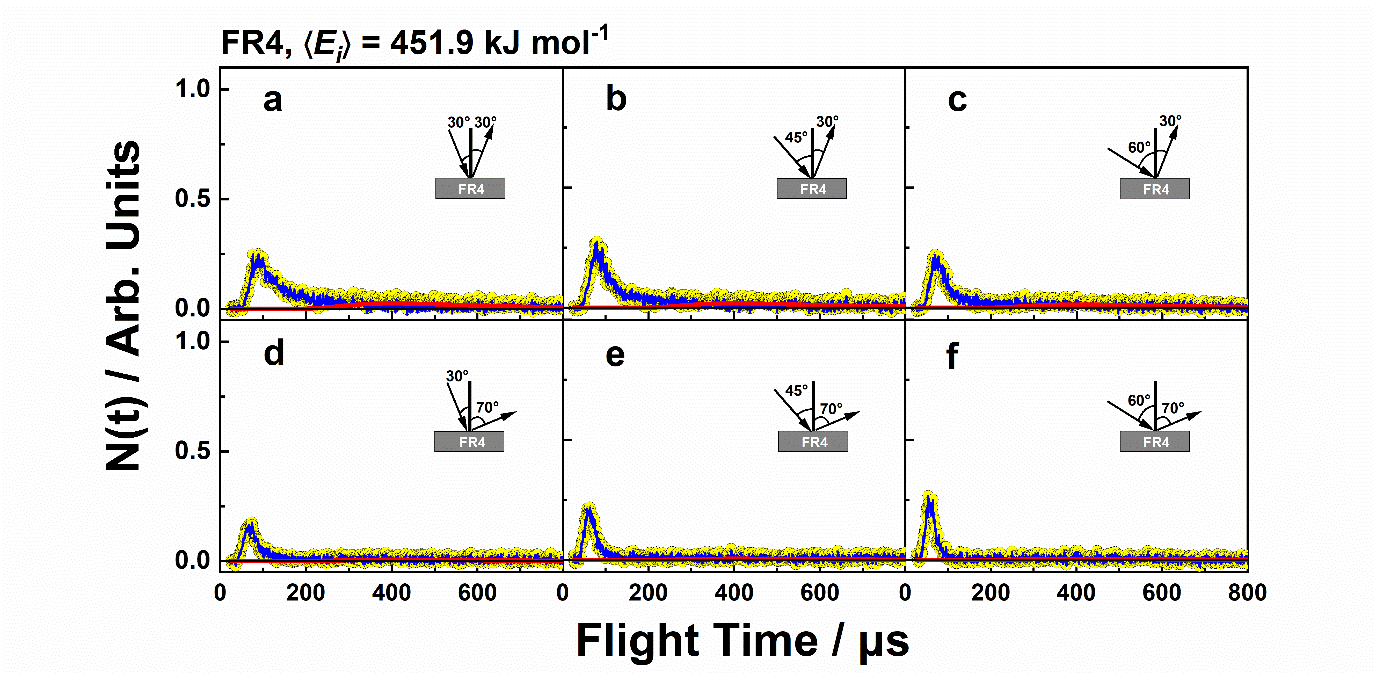


**Fig. SI3** Representative time-of-flight distributions for in-plane O-atom scattering from FR4 with an average incident energy of 〈*E_i_*〉 = 451.9 kJ mol^-1^. The experimental data are shown as yellow symbols, and the corresponding incident and final angles, *θ_i_* and *θ_f_*, respectively, are indicated in each panel. The red curves are MB fits to the TD components, and the differences between the experimental TOF distributions and the MB fits are taken to be the IS components (blue curves).


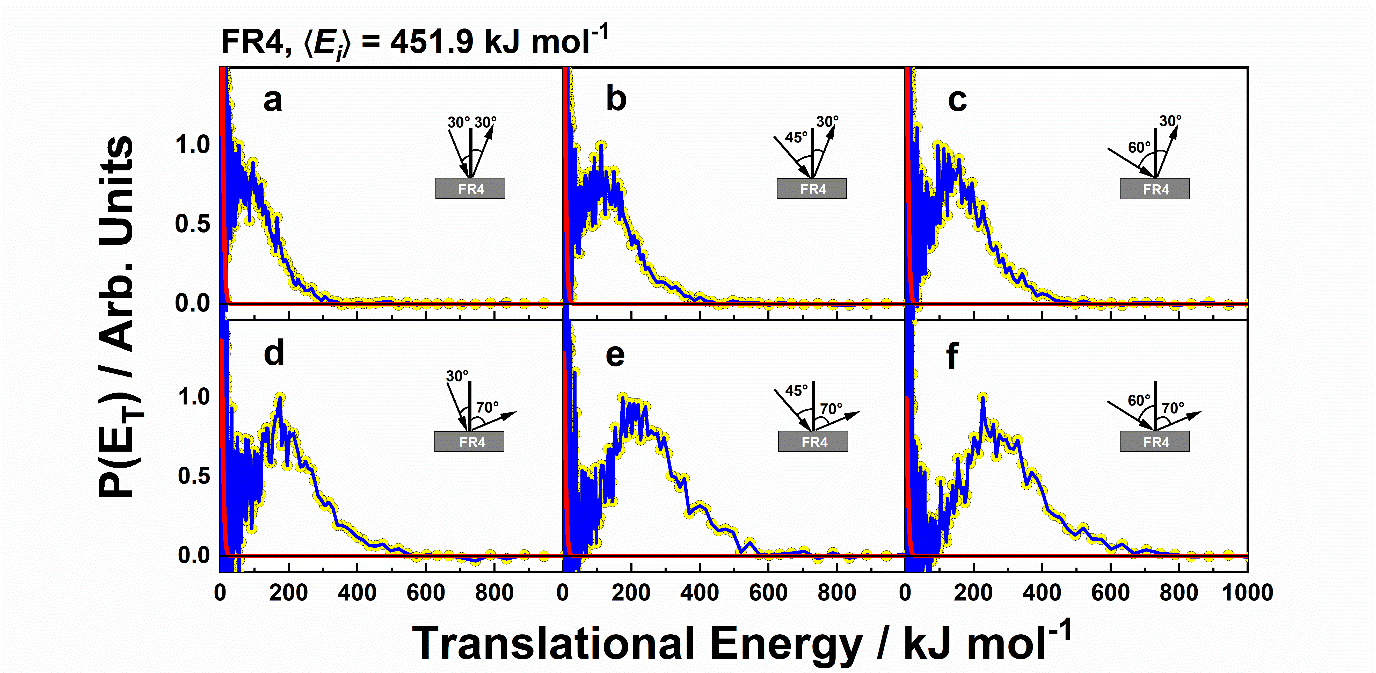


**Fig. SI4** Translational energy distributions for in-plane O-atom scattering from FR4 with an average incident energy of 〈*E_i_*〉 = 451.9 kJ mol^-1^, derived from the TOF distributions in Fig. SI1. The experimental data are shown as yellow symbols, and the corresponding incident and final angles, *θ_i_* and *θ_f_*, respectively, are indicated in each panel. The red curves are MB fits to the thermal desorption TD components, and the differences between the experimental TOF distributions and the MB fits are taken to be the IS components (blue curves). The TD flux is relatively low, and the integrals of the TD components are almost negligible compared to those of the IS components.


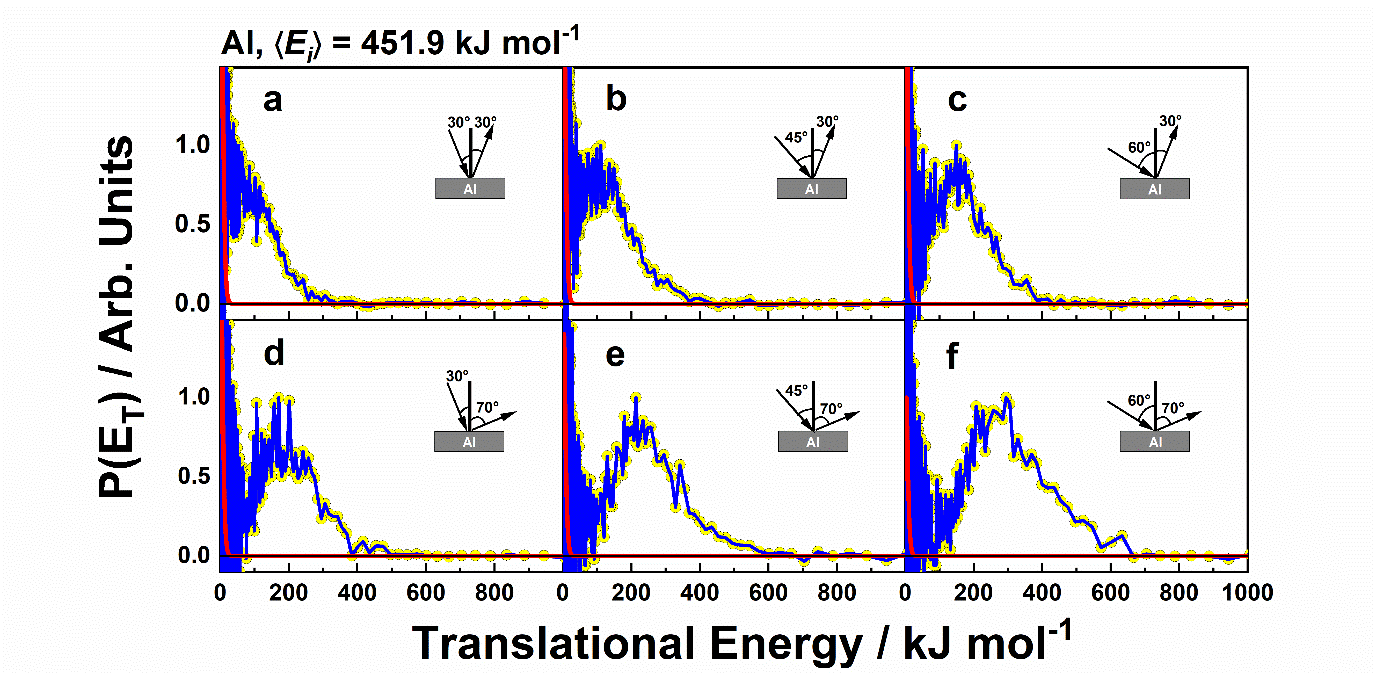


**Fig. SI6** Translational energy distributions for in-plane O-atom scattering from Al with an average incident energy of 〈*E_i_*〉 = 451.9 kJ mol^-1^, derived from the TOF distributions in Fig. SI1. The experimental data are shown as yellow symbols, and the corresponding incident and final angles, *θ_i_* and *θ_f_*, respectively, are indicated in each panel. The red curves are MB fits to the thermal desorption TD components, and the differences between the experimental TOF distributions and the MB fits are taken to be the IS components (blue curves). The TD flux is relatively low, and the integrals of the TD components are almost negligible compared to those of the IS components.


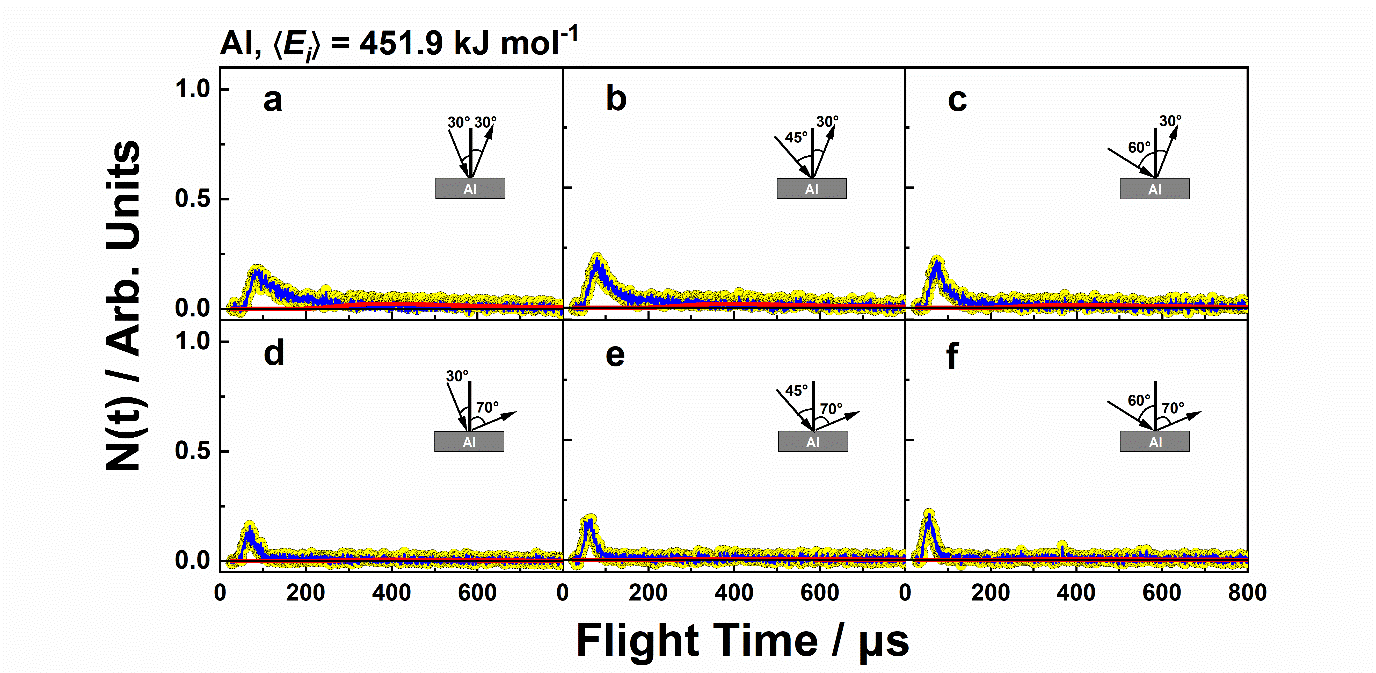


**Fig. SI5** Representative time-of-flight distributions for in-plane O-atom scattering from Al with an average incident energy of 〈*E_i_*〉 = 451.9 kJ mol^-1^. The experimental data are shown as yellow symbols, and the corresponding incident and final angles, *θ_i_* and *θ_f_*, respectively, are indicated in each panel. The red curves are MB fits to the TD components, and the differences between the experimental TOF distributions and the MB fits are taken to be the IS components (blue curves).

**Fig. SI8** Translational energy distributions for in-plane O-atom scattering from FEP with an average incident energy of 〈*E_i_*〉 = 451.9 kJ mol^-1^, derived from the TOF distributions in Fig. SI1. The experimental data are shown as yellow symbols, and the corresponding incident and final angles, *θ_i_* and *θ_f_*, respectively, are indicated in each panel. The red curves are MB fits to the thermal desorption TD components, and the differences between the experimental TOF distributions and the MB fits are taken to be the IS components (blue curves). The TD flux is relatively low, and the integrals of the TD components are almost negligible compared to those of the IS components.


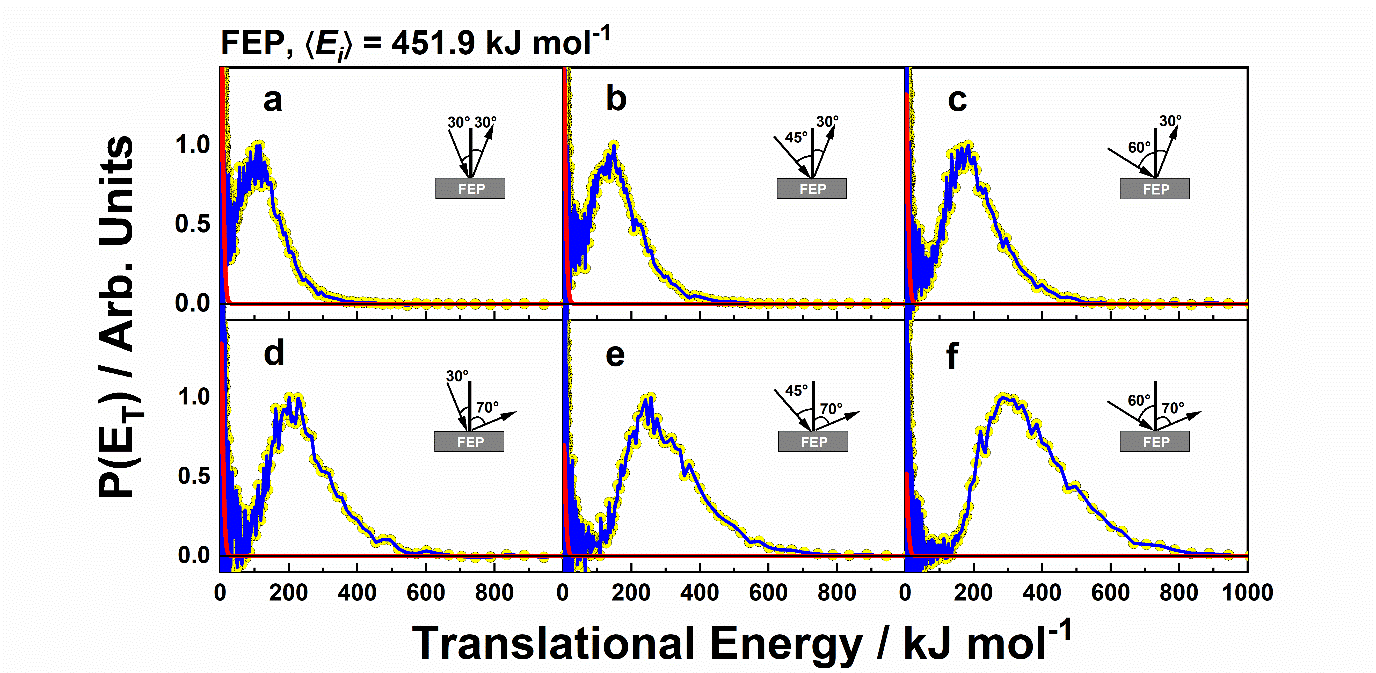


**Fig. SI7** Representative time-of-flight distributions for in-plane O-atom scattering from Al with an average incident energy of 〈*E_i_*〉 = 451.9 kJ mol^-1^. The experimental data are shown as yellow symbols, and the corresponding incident and final angles, *θ_i_* and *θ_f_*, respectively, are indicated in each panel. The red curves are MB fits to the TD components, and the differences between the experimental TOF distributions and the MB fits are taken to be the IS components (blue curves).


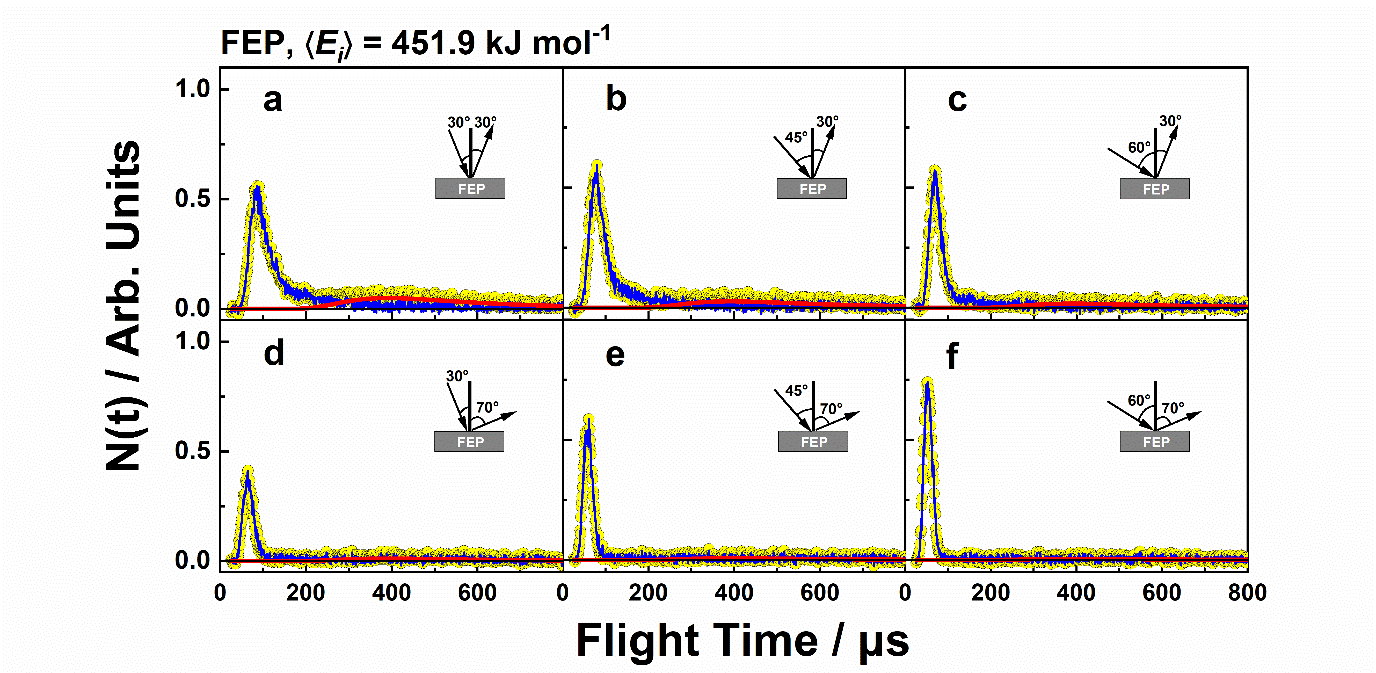


**Fig. SI9** In plane angular distributions registered at θ_i_ = 60° at m/z = 16 for O atoms that exit: (a) FR4, (b) Al, (c) CG, and (d) FEP Teflon surfaces. Yellow circles represent the total flux of O atoms scattered in the plane drawn by the incident O hyperthermal beam and surface normal, $\hat{N}$, and the blue and red circles correspond to the IS and TD components.


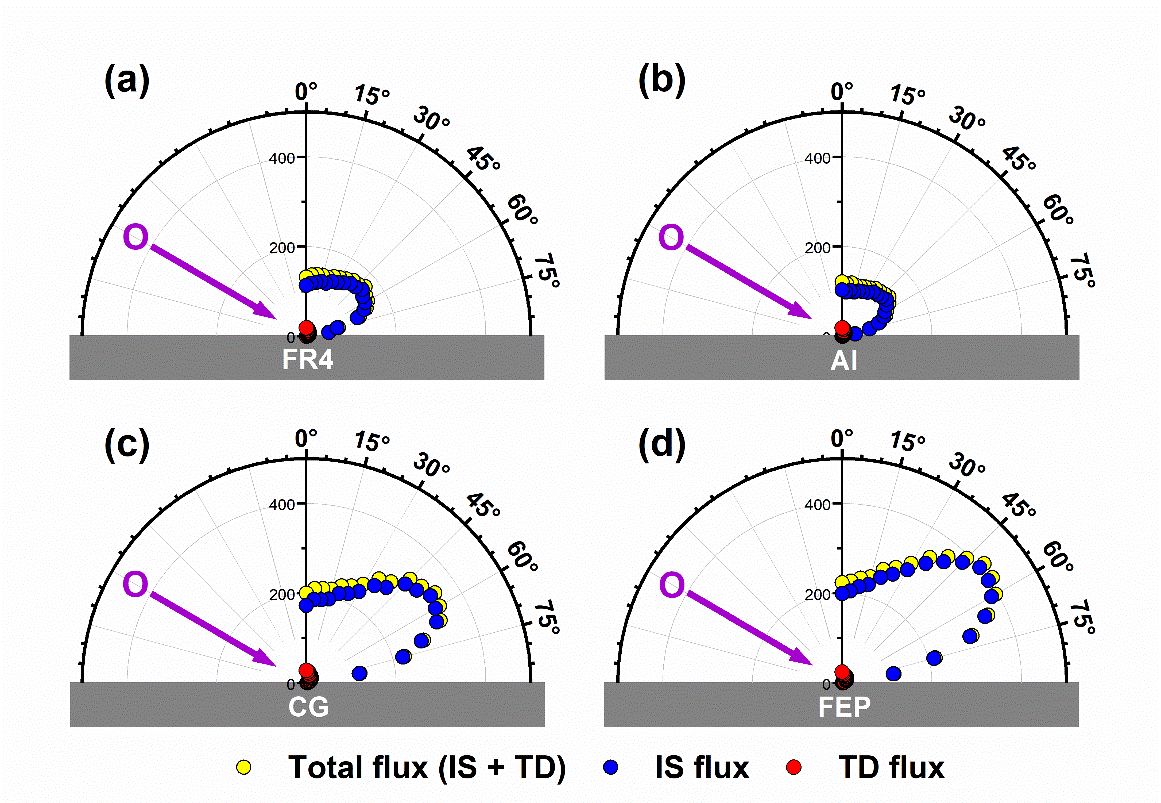

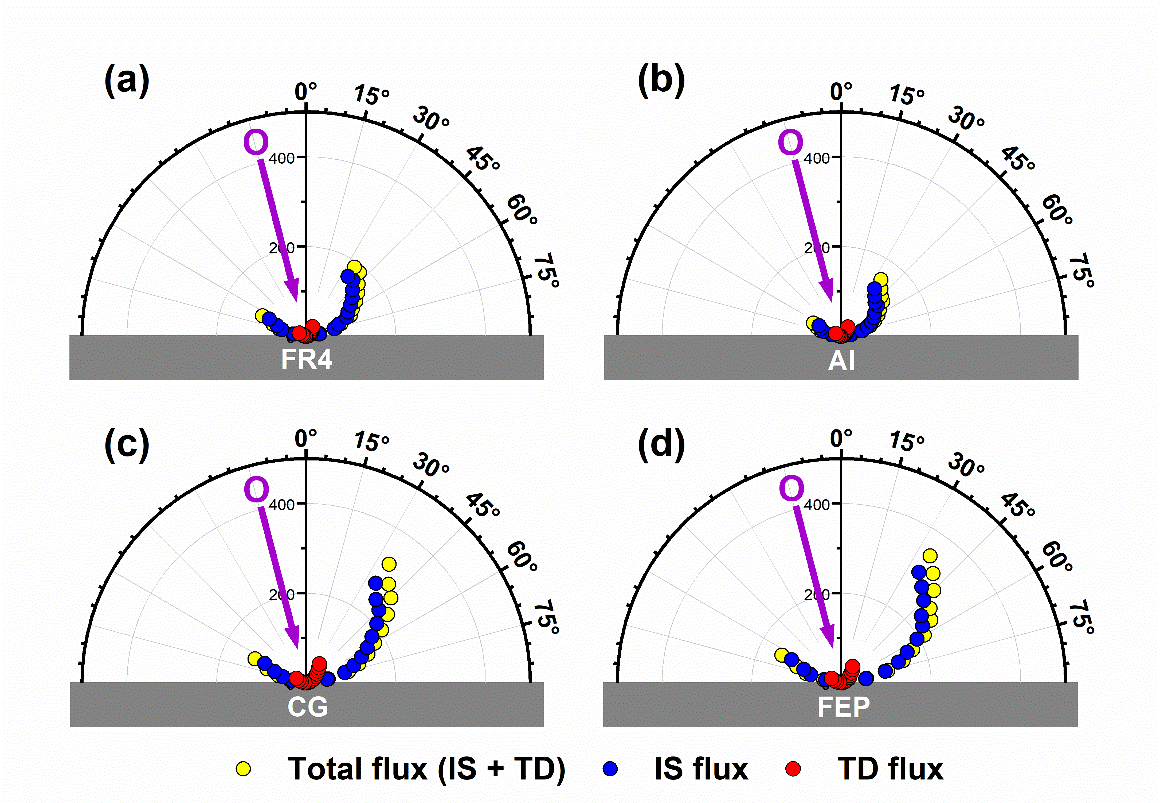


**Fig. SI10** In plane angular distributions registered at θ_i_ = 15° at m/z = 16 for O atoms that exit: (a) FR4, (b) Al, (c) CG, and (d) FEP surfaces. Yellow circles represent the total flux of O products scattered in the plane drawn by the incident O hyperthermal beam and surface normal, $\hat{N}$, and the blue and red circles correspond to the IS and TD components.

**
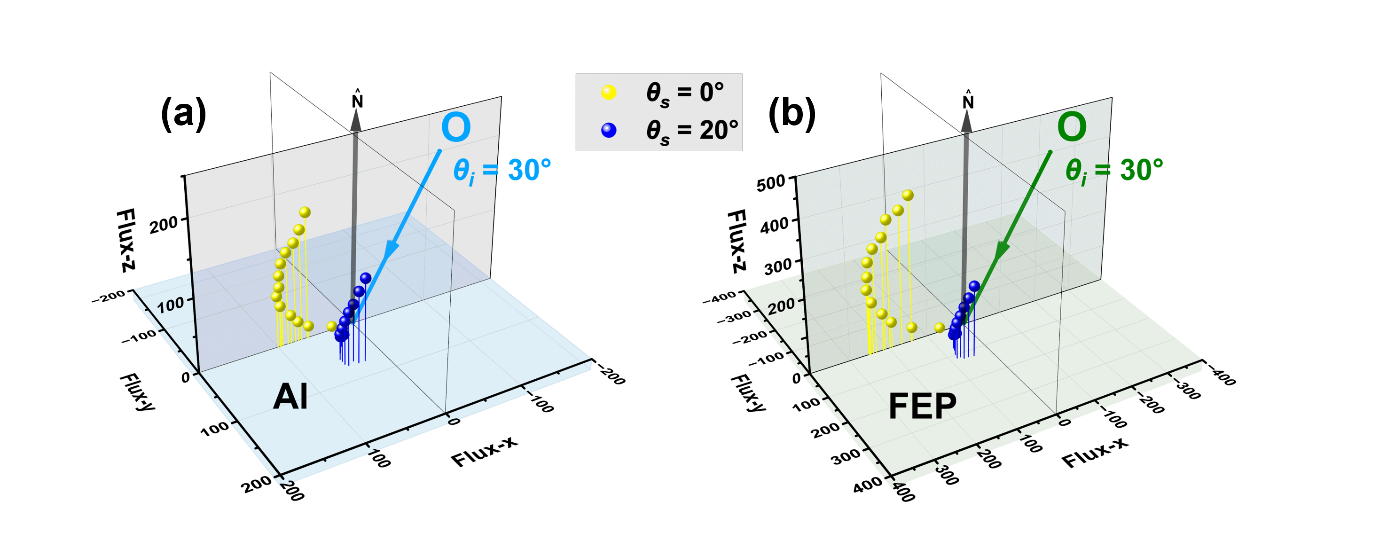
**

**Fig. SI11** Angular distributions of scattered O-atom flux from Al (a) and FEP (b) with an incident angle of 30°. Data shown correspond to sample tilt angles of 0° (yellow) and 20° (blue). Note that, with *θ_i_* = 30°, the detector rotation range did not allow product detection at a tilt angle of 45°.

1. Ann and H.J. Smead Department of Aerospace Engineering Sciences, University of Colorado, Boulder, CO 80303, United States [↑](#footnote-ref-2)
2. Space Environment Department, Soreq NRC, Yavne, 81800, Israel [↑](#footnote-ref-3)
3. Laboratory for Atmospheric and Space Physics, University of Colorado Boulder, Boulder, CO 80303, United States

   *To whom correspondence should be addressed, tminton@colorado.edu, orcid.org/0000-0003-4577-7879

   ^†^ chenbiao.xu@colorado.edu, orcid.org/0000-0002-5458-0122

   ^‡^ irina@soreq.gov.il, orcid.org/0000-0002-8219-9134 [↑](#footnote-ref-4)
